# The NLRP3 Inflammasome in Neurodegenerative Disorders: Insights from Epileptic Models

**DOI:** 10.3390/biomedicines11102825

**Published:** 2023-10-18

**Authors:** Laura Palumbo, Marianna Carinci, Annunziata Guarino, Laila Asth, Silvia Zucchini, Sonia Missiroli, Alessandro Rimessi, Paolo Pinton, Carlotta Giorgi

**Affiliations:** 1 Department of Medical Sciences, Section of Experimental Medicine, University of Ferrara, 44121 Ferrara, Italy; plmlra@unife.it (L.P.); crnmnn@unife.it (M.C.); msssno@unife.it (S.M.); rmslsn@unife.it (A.R.); pnp@unife.it (P.P.); 2 Department of Neuroscience and Rehabilitation, University of Ferrara, Via Fossato di Mortara 70, 44121 Ferrara, Italy; grnnnz@unife.it (A.G.); laila.asth@unife.it (L.A.); zcs@unife.it (S.Z.); 3 Laboratory of Technologies for Advanced Therapy (LTTA), Technopole of Ferrara, 44121 Ferrara, Italy; 4 Center of Research for Innovative Therapies in Cystic Fibrosis, University of Ferrara, 44121 Ferrara, Italy

**Keywords:** neuroinflammation, neurodegeneration, NLRP3 inflammasome, epilepsy, epileptogenesis, kainic acid, pilocarpine, glutammate

## Abstract

Neuroinflammation represents a dynamic process of defense and protection against the harmful action of infectious agents or other detrimental stimuli in the central nervous system (CNS). However, the uncontrolled regulation of this physiological process is strongly associated with serious dysfunctional neuronal issues linked to the progression of CNS disorders. Moreover, it has been widely demonstrated that neuroinflammation is linked to epilepsy, one of the most prevalent and serious brain disorders worldwide. Indeed, NLRP3, one of the most well-studied inflammasomes, is involved in the generation of epileptic seizures, events that characterize this pathological condition. In this context, several pieces of evidence have shown that the NLRP3 inflammasome plays a central role in the pathophysiology of mesial temporal lobe epilepsy (mTLE). Based on an extensive review of the literature on the role of NLRP3-dependent inflammation in epilepsy, in this review we discuss our current understanding of the connection between NLRP3 inflammasome activation and progressive neurodegeneration in epilepsy. The goal of the review is to cover as many of the various known epilepsy models as possible, providing a broad overview of the current literature. Lastly, we also propose some of the present therapeutic strategies targeting NLRP3, aiming to provide potential insights for future studies.

## 1. Introduction

### 1.1. Neuroinflammation: Concepts and Dynamics

**Figure 1:**
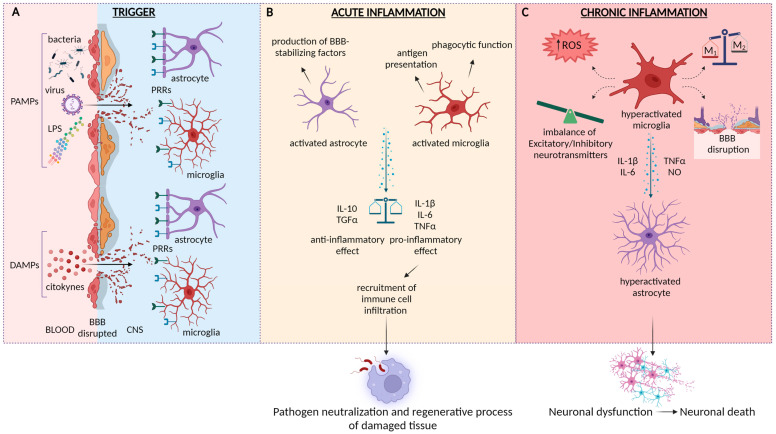
Representative diagram of the molecular mechanism underlying neuroinflammation. (**A**) Trigger phase: following the pathogen entry through the disrupted Blood–Brain Barrier (BBB), the recognition of damage-associated molecular patterns (DAMPs) and pathogen-associated molecular patterns (PAMPs) by pattern recognition receptors (PRRs) on the astrocytes and microglia surface occurs. (**B**) Acute neuroinflammation: regulated activation of glial cells. Activated astrocytes are involved in the stabilization of the BBB integrity. Additionally, they release both pro-inflammatory and anti-inflammatory cytokines in a balanced manner. Pro-inflammatory cytokines allow the recruitment of infiltrating immune system cells, while anti-inflammatory ones help maintain a regulated inflammatory state. Simultaneously, microglia activate their macrophagic and antigen-presenting functions. They also contribute, along with astrocytes, to the release of pro-inflammatory cytokines. These events lead to pathogen neutralization and activation of tissue repair mechanisms. Once their task is completed, anti-inflammatory cytokines released by astrocytes act to maintain a regulated physiological inflammatory state. (**C**) Chronic neuroinflammation: persistent and unregulated inflammatory state characterized by hyperactivation of glial cells (gliosis). Factors such as enhanced differentiation of the pro-inflammatory phenotype (M1), production of reactive oxygen species (ROS), and alteration of the balance of excitatory and inhibitory neurotransmitters by microglia contribute to neuronal dysfunction and the consequent neurodegenerative state.

Inflammation represents a responsive and safeguarding process to counteract the damaging effects of infectious agents or other detrimental triggers. The initial inflammatory response characterized by a rapid onset and short duration is referred to as “acute inflammation”; this event is considered beneficial for the host, contributing to the resolution of the problem. The process initiates with the recognition of the injury or infection by the innate immune system, triggered by the binding of pathogen-associated molecular patterns (PAMPs) or damage-associated molecular patterns (DAMPs) [1,2,3,4] to pattern recognition receptors (PRRs) on the surface of innate immune cells [5]. The activation of PRRs leads to the production of pro-inflammatory cytokines, such as tumor necrosis factor (TNF) and interleukin-1 (IL-1), which in turn lead to the recruitment of neutrophils and other immune cells to the site of damage. Once at the site, immune cells release a range of molecules, such as reactive oxygen species (ROS), nitric oxide (NO), and proteases, which are responsible for killing invading pathogens and breaking down damaged tissue. This orchestrated inflammatory response within the brain is defined as neuroinflammation and represents a fundamental physiological event capable of responding to injury or infection in the central nervous system (CNS) (Figure 1). It is now well established that CNS exploits components of the innate immune response residing in the meninges as the main surveillance mechanism, together with the presence of the blood–brain barrier (BBB) [6]; among them, the main key players are glial cells, classified as macroglia (astrocytes and oligodendrocytes) and microglia, responsible for maintaining a controlled environment essential for the structural and functional integrity of the CNS [7]. For a long time, glial cells were thought to provide only trophic support to neurons. Indeed, astrocytes, which are star-shaped cells with thousands of processes representing the most abundant glial cell type of the CNS, are involved in the formation and maintenance of the BBB [8,9,10,11], as well as in the secretion of neurotrophic factors to regulate synaptogenesis, neuronal differentiation, and survival [7,12]. On the other hand, oligodendrocytes act as physical and metabolic support to neurons by promoting the myelination process for a correct and fast signal transmission [13,14]. Lastly, microglia, the immune-competent cells of CNS, are considered the primary immune sentinels of CNS [15]. Microglia contribute to CNS homeostasis by regulating neuronal cell numbers and promoting both connectivity and synapse formation [16]. Moreover, they are involved in the stimulation of new spines in the cortex by secreting neurotrophic factors [17]. Nevertheless, the notion that glial cells solely provide trophic support to neurons has largely been challenged and overcome by the discovery of a complex network of cell–cell interactions that actively participate in immune modulation and the neuroinflammatory response [18].

#### 1.1.1. Role of Astrocytes in Neuroinflammation

As previously mentioned, astrocytes are involved in maintaining the integrity of the BBB through their interaction with endothelial cells. This function is essential for regulating the movement of peripheral immune cells into the CNS following an event of infection and subsequent disruption of the BBB. Accordingly, astrocyte depletion was associated with increased immune cell infiltration in a mouse model of experimental autoimmune encephalitis (EAE) [19]. In addition to regulating the permeability of the BBB, astrocytes, similar to microglia [20,21], have been shown to exhibit PRRs [22]. Following the recognition of pathogens by the PRRs, this type of glial cell undergoes a process of morphological and functional transformation known as astrogliosis, mainly characterized by hypertrophy of the soma and early upregulation of the intermediate filament glial fibrillary acidic protein (GFAP) and vimentin [23,24,25]. As a consequence, astrogliosis synthesizes molecules that support the tight junctions between endothelial cells or other BBB cellular components, as well as growth factors that stimulate tissue regeneration [26]; therefore, astrogliosis is involved in repairing damaged tissue. Moreover, reactive astrocytes are also engaged in the release of pro-inflammatory cytokines, chemokines, and adhesion molecules that attract immune cells to the site of damage [27,28]. All these cells, including microglia, T-cells, and other types of immune cells, are important for clearing foreign substances and cellular debris from the brain and spinal cord. Among the cytokines released, IL-1β [29], IL-6, and TNF-α have been detected. In particular, it has been demonstrated that TNF-α enhances the local inflammatory response through the recruitment of peripheral monocyte into the CNS parenchyma [30,31], while astrocyte-produced IL-1β is involved in a signaling cascade that triggers the release of vasoactive endothelial growth factor (VEGF), leading to increased BBB permeability and subsequently facilitating immune cell infiltration [32]. Importantly, once the immune response has been initiated and has fulfilled its function, astrocytes can switch from a pro-inflammatory to an anti-inflammatory phenotype, releasing cytokines such as IL-10 and transforming growth factor-beta (TGF-β) [33,34], thus reducing neuroinflammation and limiting the damage caused by a prolonged inflammatory status.

#### 1.1.2. Role of Microglia in Neuroinflammation

Although astrocytes have a fundamental role in supporting and regulating the inflammatory response, microglia are undoubtedly the primary effector of the immune response. In their resting state, microglia exhibit a small perikaryon and elongated, thin processes in the cell structure. In this condition, the role of microglia is to monitor the surrounding environment to maintain cerebral homeostasis. In addition, these cells serve as the primary defense against the entry of pathogens through the endothelial barrier [35]. Generally, trauma, infection, or alterations in neuronal activity trigger the morphological and functional transformation of microglia into an “active state”, which is characterized by a rounded amoeboid shape with an enlarged cell body and short, thick processes [36,37]. Additionally, the acquisition of increased motility allows them to reach the site of the injury. At this point, microglia begin proliferation, self-division, trigger their phagocytic action, present antigens to T-cells, and synthesize inflammatory cytokines, chemokines, ROS, NO, and trophic factors [38,39]. While these molecules exert detrimental effects on neurons at high concentrations, they exhibit a beneficial effect at low concentrations. Among the cytokines, IL-1β, IL-6, TNF-α, and interferon (IFN)-γ, as well as neuronal growth factors such as insulin-like growth factor 1, are all involved in the amplification of the inflammatory response through the recruitment and subsequent activation of infiltrating immune peripheral cells (granulocytes, monocytes, and lymphocytes) required to eliminate or contain infectious agents. Furthermore, TNF-α contributes to the activation of the nuclear factor-kappa B (NF-κB) signaling pathway, which is involved in immune response regulation. Accordingly, it has been observed that the activation of the NF-κB pathway is responsible for both the induction of pro-inflammatory gene transcription and the activation and differentiation of T cells [40]. Recent evidence has reported a correlation between NF-κB and the activation of inflammasome, a multiprotein complex crucial in the regulation of inflammation [41]. Simultaneously, IL-1β-release has been described to be involved in a positive feedback mechanism of inflammasome activation and in the NF-kB-dependent production of pro-inflammatory cytokines such as chemokine (C-C motif) ligand (CCL) CCL2, CCL20, and chemokine (C-X-C motif) ligand 2 (CXCL2) [42]. Nonetheless, a tight bidirectional correlation between astrocytes and microglia has been reported through the secretion of multiple cytokines and inflammatory mediators [43,44], demonstrating a positive feed-forward loop that sustains astrogliosis and inflammatory status. Clearly, to avoid uncontrolled and prolonged inflammatory activity that exacerbates neuronal damage, a balance between pro-inflammatory and anti-inflammatory activities is imperative. In this regard, similar to astrocytes, following the initial pro-inflammatory response aimed at promoting the killing of the invasive organism, microglia also shift into an anti-inflammatory phenotype in order to repair the damaged area [45,46]. 

Therefore, neuroinflammation is a physiological protective response of the host against infections, triggering itself to counteract pathogen invasion and, once its task is fulfilled, inhibiting its activity to facilitate regenerative processes for damage repair. However, the loss of this finely tuned regulation is detrimental to the well-being of the organism, causing severe issues of neuronal dysfunction connected to the progression of CNS pathologies. 

#### 1.1.3. NLRP3 Inflammasome: From Structure to Regulation

Inflammasome is a cytosolic multiprotein complex that becomes active in immune cells upon proinflammatory stimuli, and is also involved in regulating the immune response in the CNS [47]. Functioning as a sensor, it mediates the inflammatory response of the host organism against infectious and pathogenic insults. Inflammasome consists of three components: an upstream sensor for the detection of the danger signal, a downstream effector that proteolytically cleaves pro-inflammatory cytokines into their active forms, and the adaptor molecule recruited by the sensor protein, which is involved in binding and activating the effector protein [48]. Following activation, cytokines are released from the cell to execute the inflammatory response. 

As previously mentioned, PRR triggers both the activation and association of proteins that form the structure of the inflammasome complex. Different inflammasomes can be distinguished and classified according to the different PRRs. Moreover, among inflammasomes, the most common adapter is an apoptosis-associated speck-like protein containing a caspase recruitment domain (CARD) (ASC), which defines ASC-dependent inflammasomes. Additionally, the sensor protein also acts as a discriminator. As a result, five canonical inflammasomes have been identified: the nucleotide-binding oligomerization domain (NOD), leucine-rich repeat (LRR)-containing protein receptor (NLR) family members NLRP1, NLRP3, and NLRC4, which are activated by different types of PRRs, such as toll-like receptors (TLRs) and NLRs; the protein absent in melanoma 2 (AIM2), which is activated by cytosolic double-stranded DNA; and finally, the pyrin inflammasome which is activated by bacterial toxins [49]. The activation and subsequent assembly of inflammasome plays a pivotal role in fulfilling its main protective function. This can result in both the proteolytic maturation of interleukins and pyroptosis, a specific form of inflammatory cell death characterized by cellular lysis, the release of intracellular components, and an inflammatory response [50,51]. Among the inflammasomes, the NLRP3 inflammasome is the most extensively studied. When exposed to cellular stress, it promotes the maturation and release of the inflammatory cytokines IL-1β and IL-18, both of which contribute to immune responses and inflammation. Interestingly, it has been shown that aberrant activation of the NLRP3 inflammasome is often associated with a wide range of pathologies [52,53,54,55,56]. As a result, it is evident that a comprehensive study of the structure and functional mechanisms of NLRP3 inflammasome can provide promising insights for several therapeutic approaches. 

**Figure 2:**
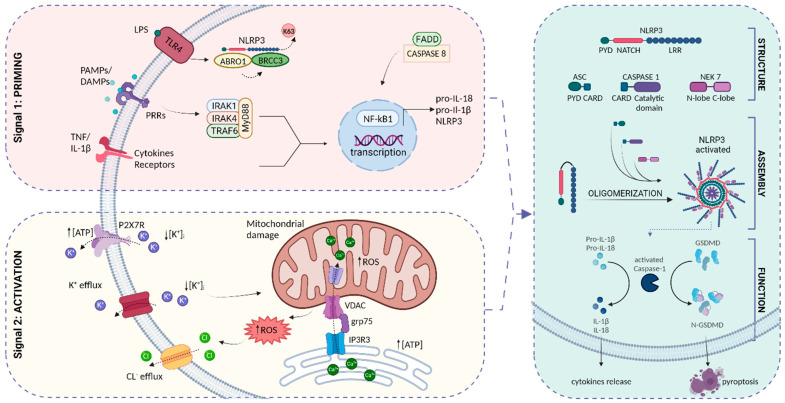
Representative diagram of the molecular mechanism underlying NLRP3 inflammasome activation. To the left of the image, the two modes of NLRP3 inflammasome activation are shown. The first signal is defined as priming, as it is an initial phase that leads to the transcription of inactive forms of NLRP3 and specific pro-inflammatory cytokines of the NLRP3 inflammasome: interleukin (IL) IL-1β and IL-18. During this phase, the activating signal arises from the recognition of DAMPs and PAMPs by the PRR receptors located on the surface of immune system cells. The second signal is defined as the actual activation phase. A reduction in intracellular potassium concentration, an increase in extracellular ATP concentration, consequent activation of the P2X7 receptor, and chloride efflux are some of the activating stimuli for the NLRP3 inflammasome. On the right side of the image, a representative schematic of the inflammasome components, assembly phase, and the function carried out by the inflammasome upon activation is shown. Once properly assembled in its activated form, the NLRP3 inflammasome facilitates the cleavage of pro-inflammatory cytokines pro-IL-1β and pro-IL-18, as well as gasdermin, into their activated forms IL-1β, IL-18, and N-gasdermin. The final event is the activation of the pyroptosis process, leading to pathogen elimination.

According to the typical inflammasome structure, the NLRP3 complex consists of three main components: the NLRP3 sensor protein, the adaptor protein ASC, and the effector protein caspase-1. NLRP3 protein is characterized by the presence of a central NACHT domain required for its activation, a C-Terminal LRR domain responsible for detecting microbial ligands, and an N-terminal pyrin domain (PYD) involved in the interaction with the ASC N-terminal pyrin domain for initiating inflammasome assembly. ASC, in turn, also contains a C-terminal CARD domain, which is responsible for the recruitment of caspase-1, a cysteine protease implicated in the secretion of pro-inflammatory cytokines [57] (Figure 2). While the structure and components of the NLRP3 inflammasome have been widely clarified over the years, the activation mechanism remains a complex process far from being understood due to its involvement in multiple steps. There are various proposed mechanisms for activation, but the most widely accepted model involves two distinct steps: priming and activation phases [58,59,60] (Figure 2). The priming step is a preparatory phase that ensures a timely and accurate inflammatory response. It is characterized by the upregulation of NLRP3 and pro IL-1β expression, triggered by the activation of various sensor proteins, including TLRs, NLRs, and cytokine receptors, through the engagement of molecules such as TNF or IL-1β. Other stimuli include the FAS-associated death domain protein (FADD) and caspase-8 [61,62]. The binding of ligands to these receptors leads to the activation of NF-κB signaling pathways, inducing the transcription of NLRP3 and pro-IL-1β at the nuclear level. One of the most well-characterized conventional stimuli is represented by lipopolysaccharide (LPS), which, through binding with the TLR4 receptor, induces the priming signal. It has been shown that LPS can activate the IL-1 receptor-associated kinases (IRAK)-1 and IRAK-4 via MyD88 [63]. An additional role for LPS has been reported, relating to the activation of the inflammasome through the regulation of the NLRP3 de/ubiquitination mechanism [64]. Indeed, the LPS–TLR4 receptor–ligand association enables the abraxas brother 1 (ABRO1) protein, which recruits the deubiquitinase BRCA1-BRCA2-containing complex 3 (BRCC3) protein that acts on the lysine (K)63 of NLRP3 [65], promoting the oligomerization and subsequent activation of the inflammasome. Other important transcriptional and post-translational mechanisms involved in the priming steps are proficiently discussed and summarized in a recent work [66]. Although transcriptional priming enhances NLRP3 activation to produce IL-1β, it requires a duration of 2 h; therefore, it is clear that the primary response of the inflammasome relies on a parallel signal. Accordingly, the use of NF-κB inhibitors failed to reduce the inflammasome activation [63]; thus, confirming that the NLRP3 inflammasome activation mechanism derives from a two-step process—following a primary trigger, a secondary activation stimulus ensues for the actual activation process (Figure 2). Among the various canonical signals proposed for the assembly and activation of NLRP3 inflammasomes, ion flow (such as potassium K^+^ efflux, Chloride Cl^−^ efflux), translocation of NLRP3 to the mitochondria, generation of mitochondrial ROS, calcium (Ca^2+^) signaling, and mitochondrial dysfunction have been proposed. The main signal for NLRP3 inflammasome activation is the disturbance of intracellular ion homeostasis. Accordingly, several studies reported that low levels of K^+^ in cytoplasm lead to the activation of NLRP3 inflammasomes [67]. The alteration in intracellular K^+^ concentration is part of a larger signaling pathway involving ATP cellular levels. In particular, an increased extracellular ATP level leads to the opening of the purinergic P2X7 receptor channels, which ultimately results in K^+^ efflux, making the P2X7 receptor one of the most potent NLRP3 inflammasome activators [68,69]. Another ion related to the activation mechanism is chloride (Cl^−^), which acts downstream of the potassium efflux-mitochondrial ROS axis [70,71]. It has been reported that NLRP3 agonists lead to potassium efflux, resulting in mitochondrial damage and the generation of ROS. Mitochondrial ROS, in turn, cause the movement of the chloride intracellular channel (CLICs) to the plasma membrane, leading to chloride efflux and promoting the interaction between NLRP3 and NIMA-related kinase 7 (NEK7), the formation of the inflammasome, caspase-1 activation, and the secretion of IL-1β [70,72,73]. Interestingly, a recent study [74] investigated the distinct roles of K^+^ efflux from that of Cl^−^ in the activation of NLRP3. Their findings revealed that K^+^ efflux is involved in the mechanism of NLRP3 oligomerization, whereas Cl^−^ efflux promotes ASC polymerization during the formation of NLRP3 inflammasome. The calcium signal has also been linked to the activation of NLRP3. This connection was established based on the observation that the Ca^2+^ chelator BAPTA-AM prevented its activation [75]. Furthermore, it has been reported that stimuli like ATP induce the mobilization of Ca^2+^ from ER to mitochondria through the mitochondrial calcium uniporter (MCU), possibly resulting in excessive mitochondrial Ca^2+^ concentration, mitochondrial dysfunction, and mtROS production [76,77]. At the same time, it was observed that the mobilization of ATP-dependent calcium leads to a weak inflow of Ca^2+^ through its receptor P2X7 while simultaneously coordinating K^+^ efflux [78]. Overall, this evidence validates that the activating signals are tightly correlated in a large flow of interactions that culminate in the assembly of the inflammasome. Accordingly, in response to the secondary signal, the NLRP3 PYD oligomerizes, acting as a scaffold for ASC nucleation through PYD–PYD interactions. Subsequently, a CARD–CARD interaction between pro-caspase-1 and ASC induces an auto-proteolytic maturation of pro-caspase-1, resulting in the formation of an active heterotetramer composed of cleaved p10 and p20 subunits. Finally, the active form of caspase-1 cleaves pro-IL-1β, pro-IL-18, and gasdermin-D (GSDMD) into their active forms IL-1β, IL-18, and gasdermin-D N-terminal, respectively [79,80]. Through this intricate activation process, the inflammasome offers the host cell a dual defense mechanism by releasing mature cytokines and removing infected or damaged cells.

### 1.2. Neuroinflammation and Neurodegeneration: A Tight Relationship

The content presented so far pertains to acute neuroinflammation, a timely regulated physiological process capable of neutralizing the pathogen and activating a regenerative process of damaged neural tissue (Figure 1B). Consequently, this mechanism is protective for the brain. However, a persistent and sustained inflammatory condition is a detrimental event leading to irreversible CNS injury [81]. Indeed, the chronic release of inflammatory cytokines and ROS can perpetuate a destructive cycle, impair neuronal plasticity, and result in cell death (Figure 1C). Accordingly, chronic glial activation significantly contributes to neurodegenerative diseases, causing neuronal dysfunction and promoting the progression of CNS disease [82]. Neurodegeneration is a complex process involving the progressive structural and functional loss of neurons in the brain [83], leading to various debilitating conditions such as Alzheimer’s disease (AD), Parkinson’s disease (PD), multiple sclerosis (MS), and epilepsy. Within the context of neurodegeneration, both microglia and astrocytes have been found to play multifaceted roles. On the one hand, they can have a protective function by removing damaged neurons and promoting tissue repair; on the other hand, when chronically activated, they can also contribute to neuronal dysfunction, exacerbating neuronal damage. Consistently, it has been reported that an imbalance in the activation of the pro-inflammatory M1 phenotype of microglia compared to the neuroprotective M2 phenotype results in excessive production and subsequent release of pro-inflammatory mediators such as IL-1β, IL-6, TNF-α, and NO [84]. Additionally, microglia have been demonstrated to contribute to neuronal dysfunction through the release of ROS and other toxic agents. These molecules can induce oxidative stress, damaging the neurons and thereby exacerbating the progression of neurodegeneration [85]. Interestingly, microglia have also been found to modulate neuronal excitability by altering the balance between excitatory and inhibitory neurotransmitters in the brain [86]. Finally, microglia have also been implicated in the disruption of BBB; the BBB is a critical structure that regulates the transport of molecules between the CNS and the bloodstream. The loss of its integrity is considered a hallmark of neuroinflammation since it can facilitate the infiltration of immune and inflammatory cells into the brain, thereby promoting the activation of other immune cells, such as astrocytes or microglia, to release additional inflammatory mediators that exacerbate neuroinflammation leading to neurodegeneration [87]. As stated before, astrocytes also contribute to the regulation of the BBB by promoting the expression of tight junction and participating in its formation. Furthermore, the release of specific cytokines establishes a positive feedback mechanism of inflammation. Indeed, it has been shown that astrocytic metabolism is influenced by IL-1β and TNF-α [88]. IL-1β has the capacity to induce a reactive state in astrocytes, promoting astrogliosis and chronic inflammation [89]. On the other hand, TNF-α up-regulates the expression of brain-derived neurotrophic factor (BDNF) in astrocytes, which mediates neuroprotective brain activity [90]. Therefore, when glial cells are activated, they can release both pro-inflammatory and anti-inflammatory factors; however, if the expression and activity of neuroinflammatory factors exceed those of the neuroprotective factors, inflammation can become detrimental to the tissues. Several studies have emphasized how chronic inflammation is triggered and sustained by characteristic markers of neurodegenerative disorders and how this mechanism can contribute to neuronal cell death in a positive feedback loop. Similarly, the accumulation of key proteins associated with diseases like Alzheimer’s or Parkinson’s has been found to activate glial cells, resulting in the release of pro-inflammatory cytokines such as IL-1β and the stimulation of the NLRP3 inflammasome pathway, leading to neurodegeneration process [91,92]. According to these investigations, in vitro models of monocyte cultures isolated from AD patients have demonstrated an upregulation in the gene expression of components of the NLRP3 inflammasome, as well as the corresponding cytokines released by this pathway [93,94]. Similarly, the involvement of NLRP3 inflammasome in PD disease progression has also been reported [91]. Increased NLRP3 expression levels, correlated with motor severity, have been observed in samples obtained from PD patients [95,96]. Additionally, higher levels of both IL-1β and IL-18 were found in the cerebrospinal fluid of PD patients compared to control subjects [97]. Interestingly, NLRP3 has also been associated with MS through the release of IL-1β and IL-18 [98], and increased mRNA levels of NLRP3 have been observed in a murine EAE model [99]. Coherently, the deficiency of NLRP3 and other components of inflammasome have been linked to a reduction in the severity of the EAE model. These findings support the notion that an altered neuroinflammatory state contributes to neurodegeneration, and the latter exacerbates neuroinflammation in the CNS. While the correlation between pathologies like Alzheimer’s, Parkinson’s, or multiple sclerosis and the role of NLRP3 has been extensively studied, unfortunately, epilepsy, a complex neurological disease, has not received the same level of attention. Therefore, the goal of this review is to highlight the role of chronic inflammation with a specific focus on the NLRP3 inflammasome in the neurodegenerative damage associated with epilepsy progression. 

### 1.3. Epilepsy

Out of all neurological diseases, epilepsy is the most prevalent condition, chronically affecting over 60 million people worldwide, regardless of age, social class, and geographic location [100]. This epidemiological scenario represents a significant portion of the global disease burden, as reported by the world health organization (WHO). Epilepsy is traditionally defined as a chronic neurological disorder characterized by recurrent seizures and unusual behavior resulting from abnormal electrical brain activity. Epileptic syndromes are typically classified into two main categories: partial and generalized. Partial seizures originate within a localized cerebral area, whereas generalized seizures occur throughout the forebrain from the outset. Several factors contribute to the onset of seizures, including increased cerebral excitatory activity, altered inhibitory regulation of excitability, and significant influences from ion regulation. Brief episodes of simultaneous excessive electrical discharges in a neuronal network of different regions of the brain occur in epilepsy, with frequencies ranging from less than one per year to several per day. As these discharges spread, they can result in loss of awareness and/or involuntary movements [101]. Epilepsy is not a single disorder but rather a wide spectrum of conditions stemming from various causes, leading to diverse neurobiological, cognitive, and psychosocial consequences. Accordingly, extensive research has shown that the development of some forms of epilepsy can be attributed to different factors, including genetic defects (e.g., polymorphisms, copy number variations, or de novo mutations), developmental dysfunction, or identifiable epileptogenic insults such as traumatic brain injury (TBI), an episode of status epilepticus (SE), stroke, or brain infection. These factors can contribute to synaptic morphological changes and hyperexcitable neuronal transmission [102,103]. These forms of epilepsy secondary to brain injury, such as temporal lobe epilepsy (TLE), can manifest months or even years after the initial insult, representing the transformation of a healthy brain into one prone to epileptic seizures. During this latency period, known as epileptogenesis, complex pathophysiological mechanisms come into play, such as neuronal death, neuroinflammation, neurogenesis, gliosis, aberrant axonal synaptic plasticity, and BBB damage [104]. All these events contribute to circuit reorganization and/or abnormal excitability [105]. Importantly, based on numerous experimental and clinical evidence, epileptogenesis extends beyond the period before the diagnosis of epilepsy with the onset of the first clinical seizure. Underlying pathological processes may persist beyond the latency period, leading to an increase in seizure frequency and severity [103,106]. The term “disease progression” is used to describe the continuation of those molecular, cellular, or network changes associated with recurrent seizures, resulting in long-term alterations in neural circuits. Additionally, epileptogenesis is often associated with comorbidities, which may arise from the involvement of common brain areas and/or the effects of spontaneous recurrent seizures (SRSs) [107]. The most prevalent histopathological abnormality found in patients with drug-resistant TLE is hippocampal sclerosis (HS), characterized by severe pyramidal cell loss and astrogliosis, primarily affecting different sectors of the Ammon’s horn (CA) [108,109]. Several well-defined animal models of TLE with HS have been developed for research purposes, and comparative studies between animals and humans have been conducted to investigate the effects of neurodegeneration and neuroinflammation during various stages of disease progression. Typically, in a murine model of TLE, chemoconvulsant administration (i.e., pilocarpine) leads to the appearance of degenerating in different brain areas, particularly in the CA1, CA3, and hilus of the dentate gyrus (DG) in the hippocampus. This hippocampal damage closely resembles human HS, a common histopathological feature of epilepsy [110]. Along with cell death in specific hippocampal areas, such as the CA3 and hilum of the DG, mossy fiber sprouting (MFS) occurs. MFS is a well-studied form of axonal plasticity in DG granule cells that has been observed in numerous experimental models [111,112] and in the hippocampus of surgical patients with various forms of epilepsy [113,114]. Indeed, during the chronic phase of the disease, MFS gradually lose their inhibitory synaptic targets, and axonal sprouting may interact with the dendrites and soma of DG granule cells, promoting the formation of an excitatory reverberating network [115,116]. Additionally, the injured epileptogenic hippocampus exhibits various indicators of inflammation, including astrocytosis, microgliosis, and IL-1β expression [117]. Neuropathological examination of surgical specimens from epilepsy patients with TLE revealed a complex and prolonged inflammatory process. In addition to astrocytes and microglial/macrophage cell activation in the hippocampus, a sustained induction of pro-inflammatory mediators, including several pro-inflammatory chemokines and cytokines, occurs [118]. 

## 2. Exploring Neuroinflammation in Epilepsy: Insights from Diverse Experimental Models

**Figure 3:**
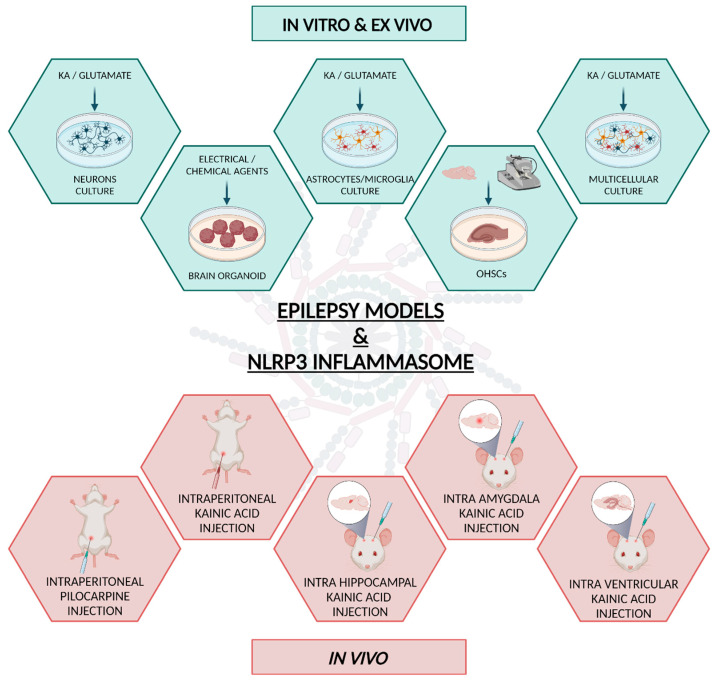
Representative schematic of epilepsy models reported in the review. At the top of the figure, the main in vitro and ex vivo models used are presented: neuronal cultures (used for the investigation of the fundamental molecular mechanisms involved in the pathogenesis of epilepsy); astrocytes and microglia culture (used for the evaluation of inflammation); multicellular culture (a more physiological model that allows the observation of cellular interactions); brain organoids and organotypic hippocampal slice cultures (OHSCs) are shown, which are more complex and suitable models for the study of the pathology due to their ability to maintain brain structure. Finally, in vivo models involve the use of pilocarpine or kainic acid (KA) administered to mice through different methods. They all represent validated and widely used models in the literature for studying the molecular events underlying the pathology and, above all, for the evaluation of parameters such as comorbidity and seizure genesis, while also identifying potential mechanisms of epileptogenesis.

Neuroinflammation may represent a crucial process associated with HS that needs to be further investigated. Accordingly, the aim of this review is to highlight the role of neuroinflammation in the neurodegenerative progression in the most well-known models of epilepsy reported in the literature, categorized within different experimental models (Figure 3). The purpose is to assess the current state of the art and identify potential novel aspects that are yet to be explored.

### 2.1. Perspective from In Vitro and Ex Vivo Models

In vitro models allow us to investigate the cellular and molecular changes that occur during epileptic seizures, including changes in ion channels, neurotransmitters, and synaptic connections. There are several types of in vitro and ex vivo models of epilepsy, including dissociated neuronal cell cultures [119,120], human pluripotent stem cells (hPSC)-based brain organoid models [121], and brain slice preparations like organotypic hippocampal slice cultures (OHSC) [122,123].

#### 2.1.1. Dissociated Neuronal Cell Culture Model

The in vitro dissociated neuronal cell culture models of epilepsy mainly involve culturing neuronal cells, typically derived from rodent brains, in a dish (Figure 3). To induce epileptic-like activity, these cells are usually treated with exogenous neurotransmitters such as glutamate, which is one of the major excitatory neurotransmitters in the brain. This procedure leads to the generation of spontaneous synchronous spiking activity among neurons, which further progresses into spontaneous seizure-like events after a distinct latency period [124]. Thus, this model facilitates the investigation of the fundamental molecular mechanisms involved in the pathogenesis of epilepsy, as well as the evaluation of drug efficacy for its treatment [119]. While reproducing epilepsy-like activity by culturing neuronal cells in a dish remains the simpler approach for in vitro epilepsy research, it is crucial to recognize that investigating the involvement of inflammation in epilepsy requires a distinct model relying on brain cells involved in the inflammatory response, including astrocytes and microglia (Figure 3). Indeed, several studies investigating the role of inflammation in epilepsy have revealed the involvement of NLRP3 inflammasome in cultured primary microglia derived from epileptic mice treated with picrotoxin (PTX) or by a BV2 model of epilepsy [125,126]. In addition, KA treatment has been reported to enhance the expression of NLRP3, cleaved-caspase-1, IL-1β, and IL-18 in astrocytes. Coherently, MCC950 (NLRP3 inhibitor) and Z-YVAD-FMK (caspase-1 inhibitor) administrations were able to abolish the effects conferred by KA [127]. Even if this evidence indicates the involvement of NLRP3 inflammasome activation in this pathological in vitro model, a more accurate reproduction of epilepsy disease for in vitro cellular simulation should require a more complex model that extends beyond culturing dissociated neurons alone, or microglia and astrocytes. 

The development of multicellular culture models could allow researchers to recreate some aspects of the cell–cell interactions that occur in the brain, providing a more complex representation of the cellular interactions that take place in the CNS and may be useful for testing potential drug candidates. Indeed, the interplay among neurons, astrocytes, and microglia plays a pivotal role in shaping neuroinflammation as a response to insults in the CNS. Thus, although such models are important for achieving a deeper understanding of how cellular communication influences the process of neuroinflammation and, concurrently, how it affects neurons in pathological conditions like epilepsy [128,129], they present some limitations since they cannot fully capture the complexity of the brain.

#### 2.1.2. hPSC-Based Brain Organoid Models

In recent years, another interesting model that could be used to study the role of inflammation in epilepsy has been represented by cerebral organoids (Figure 3) [130], which are emerging as a groundbreaking technology for studying various aspects of human brain development and disorders in a three-dimensional culture system and offer a hopeful approach for simulating diseases [131]. Cerebral organoids are usually generated from hPSCs, which can differentiate into the various cell types found in the brain [132,133,134]. By allowing these cells to self-organize in a three-dimensional structure, it is possible to replicate aspects of brain organization that would be impossible to study in traditional two-dimensional cell cultures. According to the literature, depending on the specific protocol used, cerebral organoids can mimic different regions of the brain, offering insights into the development of various CNS components such as the hippocampus, midbrain, hypothalamus, cerebellum, thalamus, anterior pituitary, and retina [135,136,137,138,139,140,141,142]. These human cell-based models have multiple significant aspects as they are versatile and capable of becoming various cell types, offering a hopeful approach for simulating diseases, testing, and creating drugs, as well as evaluating a drug’s potential toxic effects. Brain organoids can be stimulated with electrical or chemical agents to induce epileptic-like activity, allowing for the investigation of epilepsy in a more realistic and complex brain environment than traditional in vitro models. When considering the functioning of neurons, these organoids can be cultured for more extended periods in comparison to two-dimensional cultures, resulting in a higher level of maturation not only among the neurons but also in the astrocytes [143]. In addition, induced pluripotent stem cells (iPSCs) that originate from patients have identical genetic mutations within the unique genetic context of each patient. Since it is impossible to access brain tissues for laboratory study from a person with a CNS disorder, guiding the transformation of hPSCs into neural cells or brain-like structures presents an unmatched chance to simulate the progression and disease of the human brain. Moreover, developing organoids that naturally incorporate microglial cells has proven to be a challenging task. Indeed, a limitation of CNS organoid protocols is their tendency to push cells toward the neuroectoderm lineage while inhibiting the formation of mesoderm and endoderm. As a result, CNS organoids have been observed to lack the comprehensive array of cells originating from various germ layers found in the brain in vivo, including microglia [144,145]. However, recent research has introduced innovative approaches for generating organoids containing cells from all germinal layers, including the microglia [146,147,148,149,150,151,152]. This combination offers enhanced insights into the mechanisms underlying neurodegenerative disorders, including epilepsy. Thus, although these techniques offer exciting opportunities for studying human brain development and disorders, including the intricate cell–cell communication network responsible for the mechanisms underlying epilepsy [153,154,155], they also come with challenges and limitations. These involve the intricate nature of the organoids, their incomplete development compared to a brain, and the necessity to enhance the reproducibility and uniformity of the methods. Despite these obstacles, the field is progressing rapidly, and cerebral organoids are able to deepen our understanding of the human brain and its disorders.

Even though they have inherent limitations, in vitro cell culture models of epilepsy have proven to be valuable tools for investigating the cellular and molecular mechanisms underlying epileptic activity, representing a relatively simple and easy-to-use platform for investigating brain diseases like epilepsy and for testing potential drug candidates. Future research should aim to develop more advanced in vitro models of epilepsy to study the role of neuroinflammation that captures the complexity of the brain and its interactions with other organs while maintaining the simplicity and ease of use of traditional in vitro models. In this regard, the use of organotypic hippocampal slice cultures can be considered as an alternative in vitro model to study the inflammatory aspects of epilepsy, given their ability to maintain brain structure and connectivity [156].

#### 2.1.3. Organotypic Hippocampal Slice Cultures

Although producing valuable results, ex vivo models have not been broadly used to investigate the role of neuroinflammation-NLRP3 dependent on epilepsy. However, few studies using organotypic hippocampal slice cultures (OHSC) have shown interesting results (Figure 3). OHSC maintains some of the intrinsic properties of the tissue, including the most important aspects of connectivity and neuron–glia interactions [157]. Different from acute hippocampal slices, OHSC conserve the three-dimensional cytoarchitecture of the brain [158] and allows the reproduction of both latency and chronic epileptic conditions. In fact, OHSC displays interictal-like spikes preceding long-lasting clusters of ictal-like discharges, which increase over time and are sensitive to antiseizure treatment [159,160]. Detailed characterization of OHSC has highlighted them as an ex vivo model that mimics key features of clinical and experimental in vivo epilepsy and enables the investigation of antiseizure and anti-epileptogenic drugs [161]. OHSC has also been recently suggested as a model of NLRP3 inflammasome activation in microglia, highlighting NLRP3 inflammasome relevance in neuroinflammation [156]. As far as the hole of NLRP3-dependent neuroinflammation on epilepsy is concerned, NLRP3 over-expression and activation were found in OHSC with epileptiform activity when compared to control [122]. Other noticeable features of neuroinflammation found to be related to epileptic-like activity in OHSC were the upregulation of pro-inflammatory cytokines (i.e., IL-1β, IL-6, and TNFα), activation of astrocytes and microglia [122,162], and increased neuronal death. The authors suggested that NLRP3 inflammasome may be involved in pyroptosis-induced neuronal death occurring during epileptogenesis [122], although cell-death mechanisms involved in the OHSC model of epilepsy have not yet been clarified. Although Shaker and co-workers could not define the enhanced neuronal excitability found in pyramidal neurons in an OHSC as epileptic-like activity, they have shown that peripheral induction of NLRP3 inflammasomes could elicit pathophysiological changes in brain excitability. They have co-cultured activated spleen-derived peripheral blood mononuclear cells (after triggering inflammasome formation) with OHSC and suggested that hyperexcitability in the hippocampus is mediated by NLRP3 stimulation followed by up-regulation of IL-1α and IL-1β, caspase-1 activation, and the consequent decrease of K^+^ currents that would dampen neuronal membrane excitability [163]. In line with these results, the treatment of OHSC with curcumin, an anti-inflammatory and potent inhibitor of NLRP3 inflammasome activation [164], decreased the number of neurons displaying seizure-like activity, even if curcumin was not able to reduce the increased number of astrocytes nor the increased gene expression of the inflammatory markers IL-1β, IL-6, and TGF-β found in the hippocampal slices [165]. Taken together, these results suggest that NLRP3-dependent neuroinflammation may play a role in epilepsy through the involvement in hyperexcitability of the hippocampus. However, further studies are needed to pursue the open questions, and the OHSC ex vivo model may be helpful in this regard. Since OHSC can maintain neuron-glia interactions, it is possible to characterize the NLRP3 inflammasome cascades in hippocampal cells under a controlled environment. It is of special importance to understand how each cell and tissue can regulate NLRP3 activation through different mechanisms [166]. Pharmacological manipulations of NLRP3 and its up-/downstream factors through the application of antagonists in OHSC cultures may help to identify the specific signaling pathway involved in epileptic-like activity. Lastly, the treatment of OHSC cultures with validated anti-inflammatory drugs could support their use in the treatment of epilepsy. 

### 2.2. Investigating In Vivo Epilepsy Models

It is known that epileptogenesis is associated with severe and chronic neuroinflammation, accompanied by neuronal damage and gliosis [167]. All these molecular and cellular events work in a loop in which seizures induce inflammation and inflammation, that, in turn, exacerbates seizures, contributing to the progression of the disease. The role of NLRP3 in epilepsy has been investigated in vivo preclinical studies using various animal models (Figure 3). Indeed, compared to in vitro models, the use of animal models offers the advantage of better modeling of a complex disease such as epilepsy, thus offering the possibility of better studying the pathogenetic mechanisms underlying the disease. In the specific case of epilepsy, the in vivo model allows for the evaluation of parameters such as comorbidity and seizure occurrence and also identifies potential mechanisms of epileptogenesis. However, the creation of such a complex model can be more challenging due to the greater number of parameters that need to be considered. In this regard, the choice of certain substances over others, as well as the different methods of administration, can affect both the success of the experimental model and the outcome of the analysis.

The first evidence of the correlation between NLRP3 and epilepsy in an in vivo model emerged from a study conducted in 2014, aiming to explore the role of NLRP3 inflammasome in neuroinflammation, spontaneous recurrent seizures, and hippocampal neuronal loss in a rat model of amygdala kindling-induced SE. The authors observed an increase in cleaved IL-1β levels and NLRP3 inflammasome components beginning at 3 h after the onset of SE and reaching their peak at 12 h. Knocking down of NLRP3 or caspase-1 resulted in significant suppression of the development and severity of SRS during the chronic epileptic phase, along with a remarkable reduction in hippocampal neuronal loss in the CA1 and CA3 areas 6 weeks after SE [168]. Treatment with natural antioxidants such as amentoflavone and biochanin A reduced seizure susceptibility in pentylenetetrazole (PTZ)-induced kindling mice. This reduction was achieved by inhibiting PTZ-induced expression of NLRP3, ASC, and caspase-1, thereby blocking neuronal apoptosis and improving cognitive performance [125,169]. Moreover, ibuprofen, a non-steroidal anti-inflammatory drug, also inhibited the expression of NLRP3, caspase-1, and IL-18 in a chronic model of PTZ-kindled epileptic rats. In comparison with the PTZ control group, PTZ animals treated with ibuprofen showed significantly fewer SRSs and less damage to hippocampal neurons [170]. All this evidence demonstrates a strong correlation between NLRP3-dependent neuroinflammation, inducing neurodegeneration, and epilepsy progression. In order to further investigate the involvement of the NLRP3 inflammasome in the epileptic process, the following sections will discuss two main in vivo epileptic murine models obtained by the administration of two chemo-convulsants: pilocarpine and kainic acid (KA) (Figure 3). 

#### 2.2.1. Pilocarpine Model

In a murine model of epilepsy induced by intraperitoneal (i.p.) injection of pilocarpine in wild-type (WT) mice, increased expression levels of IL-1β and NLRP3 were observed in the peripheral blood and hippocampus, 3 days after SE, while the decrease identified at 7 days remained higher than in the control untreated group. Moreover, at day 7, the levels of neuronal necrosis and apoptosis in the hippocampal CA3 region of WT mice were higher compared to the untreated mice. IL-1β levels in the peripheral blood and cell damage were also measured in NLRP3 KO mice treated with pilocarpine, being lower than in the WT group 3 days after SE. These results suggest that NLRP3 may be involved in the development of refractory TLE [171].

Notably, the findings from Wang and colleagues [172] confirmed that pilocarpine administration can induce the activation of the NLRP3 inflammasome pathway in mice. Their results also showed an enhancement of neuronal death and an increase in gliosis. In addition, it has been demonstrated that signal transduction activator of transcription 3 (Stat3), highly expressed in the hippocampus of epileptic mice treated with lithium-pilocarpine, binds to the NLRP3 promoter enhancing H3K9 acetylation, NLRP3 transcription, and NLRP3/caspase-1-mediated neuronal pyroptosis, resulting in worsening of neuronal damage in epileptic mice [173]. The circadian nuclear receptor Rev-Erbα regulates the expression of NLRP3, the secretion of cytokines by macrophages, and acts as a significant negative regulator of neuroinflammation. Surprisingly, it was found to be reduced in both the early post-SE and chronic phases in a pilocarpine mouse model. Consistently, the treatment with SR9009 (Rev-Erbα agonist) for 7 days after SE reduced SE-related inflammation NLRP3-dependent and neuronal damage, suggesting that the downregulation of Rev-Erbα exacerbates neuronal apoptosis [174]. Transient receptor potential melastatin 2 (TRPM2) is a non-selective calcium channel that has been implicated in exacerbating brain injuries associated with epilepsy. TRPM2-KO mice treated with i.p. pilocarpine administration exhibit mild neuroinflammation with reduced mRNA production of IL-1β, TNF-α, CXCL2, and IL-6, as well as lower levels of NLRP3, ASC, and caspase-1 protein expression and glial activation compared to WT mice. Moreover, TRPM2-KO mice displayed low neurodegeneration and improved cognitive performance [175], confirming the role of NLRP3-dependent inflammation in the development of epileptic disease. 

It has been shown that eugenol, the primary phytoconstituent of essential oils, reduced apoptotic neuronal cell death induced by pilocarpine, mitigated both astrocytosis and microgliosis, attenuated the expression of IL-1β and TNFα, inhibited NF-κB activation, and the formation of the NLRP3 inflammasome following the onset of SE [176]. In a similar study, NLRP3 inflammasome components were activated upon lithium-pilocarpine induction in immature rats. Notably, the administration of gentiopicroside (Gent), a natural herb isolated from Gentianaceae, 18 h before inducing, led to a reduction in seizures susceptibility and an improvement in cognitive functions. Moreover, these animals displayed decreased neurodegeneration and reduced pro-inflammatory cytokines levels, as well as a diminished expression of P2X7R. Consistently, Gent and P2X7R inhibitor administration suppressed the activation of NLRP3 inflammasome in the hippocampi of epileptic immature rats, confirming the relationship between NLRP3 inflammasome and epilepsy progression [177]. 

#### 2.2.2. Kainic Acid (KA) Model

Recently, the involvement of NLRP3 has also been investigated in epileptic models induced by KA administration. I.p. repeated low doses (RLD) of KA in rats resulted in a significant up-regulation of IL-1β, NLRP3, and active caspase-1 levels. Additionally, microgliosis and neuronal loss were predominantly observed in the hippocampal CA1 and CA3 regions 8 days after inducing SE, when the animals exhibited pronounced cognitive impairments. Interestingly, pre-treatment with curcumin, a potent NLRP3 suppressor (as indicated by reference [178]), administered 7 days before KA injection, led to a significant decrease in protein levels of IL-1β, NLRP3, and cleaved caspase-1 within the hippocampus. This treatment also limited neurodegeneration within the CA3 area and mitigated KA-induced deficits in spatial learning and memory [179]. Similar outcomes were observed using the same epileptic model induced by a single i.p. injection of KA. This study aimed to evaluate the effects of neferine, an alkaloid derived from lotus seed embryos [180], as a treatment. Additionally, intra-cerebral injections, which can be categorized as intraventricular, intrahippocampal, and intra-amygdaloid, of KA demonstrated the up-regulation of NLRP3 inflammasome components. A recent study highlights the use of a therapeutic combination consisting of valproic acid and furosemide [181] for regulating the NLRP3 inflammasome complex in an epileptic rat model induced by intraventricular KA administration. The combination treatment significantly reduced the increase in cell apoptosis and elevated levels of NLRP3 and ASC observed after the induction of epilepsy. The G protein-coupled receptor 120 (GPR120) also plays a role in regulating neuroinflammation, exhibiting a protective effect in epilepsy through the NLRP3-dependent signaling [182]. The contribution of NLRP3 inflammasome in epilepsy severity is also evident in a mouse model of intrahippocampal KA administration [183].

Given its role in activating the NLRP3 inflammasome, studies have also highlighted the involvement of P2X7R in the progression of epilepsy in in vivo KA-induced models. Intriguingly, the administration of a P2X7R inhibitor to mice 11 days after inducing SE through intra-amygdala KA administration led to a reduction in spontaneous seizures and an improvement in gliosis status [184].

To date, the available literature does not provide evidence establishing a connection of NLRP3 in models of epilepsy induced by intranasal administration of KA. However, microglial activation and neuronal damage are strongly associated with this method [185]. Although various animal models have demonstrated the involvement of NLRP3 in temporal lobe epilepsy, TLE models exhibit distinct structural and functional characteristics based on the convulsant used. A study conducted in 2022 aimed to understand the molecular dynamics of hippocampal inflammasome activation resulting from systemic pilocarpine administration versus unilateral intrahippocampal KA-induced SE in mice [186]. It revealed that the transcription of NLRP3 and associated inflammasome signaling molecules occurs in a model-specific and time-dependent manner. Nevertheless, the consistent correlation reported between NLRP3-dependent neuroinflammation and the progression of neurodegeneration in various epileptic models supports the evidence presented. 

### 2.3. Epilepsy Studies Using Human Samples

The purpose of creating in vivo and in vitro models has always been to reproduce in an "artificial" and as consistent as possible what occurs in human beings during diseases. For this reason, the evaluation of molecular events that characterize a specific pathological condition directly in human samples collected from patients represents the gold standard of study methods. While evaluating NLRP3 involvement in more natural models would be desirable and preferable over artificial ones, the challenge of obtaining samples poses a tangible obstacle. Accordingly, the exploration of NLRP3 involvement in human mTLE remains poorly investigated. Nonetheless, recent research demonstrated an upregulation of the NLRP3 inflammasome markers in neurons, astrocytes, and microglia of the temporal neocortices of TLE patients [187]. Furthermore, sclerotic hippocampi from mTLE patients exhibited pronounced cytoplasmic and nuclear immunostaining for NLRP3 and IL-1β, particularly in pyramidal neurons of the CA2, granule cells of the DG, and glial cells of the CA and the stratum radiatum when compared to control autoptic samples. Noteworthy, plasma levels of IL-1β in patients with mTLE were increased compared to the controls. This result is in line with a persistent low-grade chronic inflammation. Interestingly, patients with bilateral HS and a history of febrile seizures displayed higher caspase-1 levels, underscoring a link between the severity of hippocampal damage and peripheral immunity [188]. Additionally, the findings of Wu and colleagues [171] revealed glial cell proliferation and NLRP3 expression in the cortex of patients with refractory TLE, as well as reported by Ravizza, which indicates an upregulation of IL-1β in neurons and glial cells within epileptic foci of patients [189]; therefore, recurrent epileptic seizures result in NLRP3 upregulation in microglia [190]. Additionally, elevated IL-1β serum levels in patients with intractable epilepsy were found to be correlated with the duration of an individual convulsion but not with the duration of the disease itself [171]. Thus, the levels of this inflammatory cytokine during the acute stage of a convulsion could potentially reflect the severity of the crisis. Interestingly, Song and colleagues reported an increase in P2X7R levels in patients with intractable TLE. Immunostaining for the expression of GLU and GFAP exhibited upregulation in the temporal lobes of TLE patients when compared to the control autoptic group. This observation suggests that the P2X7 receptor, recognized as one of the fundamental activators of NLRP3, might contribute to the pathology through an astrocyte-released GLU-dependent mechanism [191]. It is worth mentioning that noticeable differences in neuropathological features exist when comparing HS-TLE, which is characterized by more prominent neuronal cell loss and astrogliosis, with lesion-associated TLE, which exhibits a milder condition. However, the immunolabeling of NLRP3 shows a comparable expression pattern in both TLE pathologies as assessed by morphological analysis, suggesting that in chronic human TLE hippocampi, there is a robust and largely indistinguishable expression of key inflammasome components regardless of the neurodegenerative state and the extent of astrogliosis [186]. 

## 3. NLRP3 Inhibition: A Promising Strategy for Epilepsy Treatment

There is a wide range of anti-seizure medications available for treating epilepsy, each with distinct mechanisms of action and effects (Table 1). The majority of these drugs work by enhancing GABAergic activity or diminishing glutamatergic neurotransmission. Their effects are achieved by regulating voltage-gated ion channels, synaptic release machinery, or through multiple mechanisms [192]. Nevertheless, approximately 30% of all epilepsy patients exhibit resistance to these treatments [193]. As a result, extensive efforts are being directed to discover new treatments or biomarkers that can be targeted by medications. Given the evident role of neuroinflammation in various stages of epilepsy, including epileptogenesis and the chronic period, several investigations focused on exploring the potential anti-seizure effects of anti-inflammatory drugs, as well as the anti-inflammatory effects of anti-seizure medications. Indeed, certain anti-seizure medications with established clinical use demonstrated their ability to modulate the neuroinflammatory process. In a study involving rats subjected to KA-induced seizures, the combined treatment of valproate and furosemide led to reduced seizure intensity, along with a decrease in NLRP1 and NLRP3 mRNA expression in brain samples [181]. Similarly, rufinamide, a medication primarily used in pediatric epilepsies [192], was found to inhibit microglial overactivation, mitigate the neuroinflammatory response, and counteract the disruption of the BBB induced by KA in mice [194]. Likewise, the anti-epileptic effects of anti-inflammatory drugs in clinical use have also been documented in the existing literature. Ibuprofen, recognized as one of the safest non-steroidal anti-inflammatory medications, exhibited anti-epileptic effects, as indicated by reduced seizure scores and neuroprotective properties, minimizing the loss of hippocampal neurons when administered to rats exposed to the pentylenetetrazol-induced epilepsy model. While ibuprofen reduced neuronal excitability in epileptic animals, its impact on the COX-2/NLRP3/IL-18 pathway may also contribute to its anti-epileptic effect. Indeed, ibuprofen led to a reduction in COX-2 secretion, inhibited NLRP3 activation, and decreased IL-18 secretion in epileptic animals [170]. Several other anti-inflammatory compounds, exclusively studied in preclinical settings, have been investigated to elucidate the mechanisms underlying the effects of anti-inflammatory molecules on epileptogenesis and seizure parameters [195]. Notably, these studies provide the starting point for the development of clinical trials exploring anti-inflammatory treatments for epilepsy. Currently, a phase II clinical trial assessing the compound VX-765, an IL-1β synthesis inhibitor targeting caspase-1, in patients diagnosed with drug-resistant focal onset epilepsy is in progress [196]. Although there are several potential inflammatory targets, NLRP3 inflammasomes may play a pivotal role in modulating IL-1β production [197]. 

Accordingly, given the promising potential of targeting NLRP3 for the development of anti-epileptic therapies, in recent years, several companies have displayed interest in developing compounds targeting NLRP3 [52,65,198]. Due to the complex signaling cascade of the NLRP3 inflammasome, several targets can be identified to directly or indirectly inhibit NLRP3 activity [199,200]. However, it is important to emphasize that employing specific NLRP3 inhibitors represents the optimal therapeutic approach for treating NLRP3-related conditions, such as epilepsy. The relevance of NLRP3 inhibition has been highlighted by several strategies, including molecular, pharmacological, and natural approaches. NLRP3 silencing through the use of siRNA improved neuronal survival and mitigated seizure severity in an in vivo TLE model [168]. Among natural compounds, amentoflavone, a natural bioflavonoid with anti-inflammatory properties, decreased seizure intensity and cognitive impairment by inhibiting the NLRP3 inflammasome [125]. Furthermore, melatonin, a widespread hormone known for alleviating NLRP3 inflammasome activity [201], displayed a neuroprotective role in a rat epilepsy model if conjugated with sodium valproate [202]. Additionally, heightened hippocampal expression of NLRP3 inflammasome components and microgliosis were mitigated using the NLRP3 inhibitor curcumin in an epileptic rat model [179]. Regarding the pharmacological approach, the impact of CY-09 has been explored in a PTZ-induced animal model. It was reported that CY-09 improved PTZ-induced neuronal loss, lessened astrocyte activation, and reduced IL-1β and IL-18 secretion [203]. Finally, MCC950, considered one of the most potent and selective NLRP3 inflammasome inhibitors, has been recognized in the literature as a tumor suppressor factor [52]. Shen and colleagues demonstrated that exposure to MCC950 significantly ameliorated neuronal loss in both in vitro and in vivo epileptic models [204]. Intriguingly, since a recent study highlighted the role of the promyelocytic leukemia protein (PML) as a modulator of the tumor immune microenvironment, acting by inhibiting the P2X7-NLRP3 axis interaction [205], it could be valuable to explore the role of this protein in the context of epilepsy as well. Therefore, based on the strong correlation between the inflammatory NLRP3-dependent state and the progression of neurodegeneration in epilepsy, NLRP3 inhibitor compounds could be used in clinical trials for the development of a therapy aimed at reducing seizures and having a disease-modifying effect. 

**Table 1:** Beneficial effects of different compounds administrated in several epileptic models.

Compound	Effect	Results	Model
Valproate and Furosemide [181]	Anti-epileptic and diuretic effect	Reduced seizure intensity; decreased NLRP1 and NLRP3 mRNA expression in brain samples	Single dose of KA injection in adult male Wistar rats
Rufinamide [192]	Anti-epileptic effect	Inhibited microglial overactivation; mitigated neuroinflammatory response, and reduced BBB damage	Intraperitoneal injection of KA in male ICR mice
Ibuprofen [170]	Anti-inflammatory effect	Reduced seizure scores; decreased loss of hippocampal neurons	Rats exposed to the pentylenetetrazol-induced epilepsy chronic model
VX-765 [196]	Selective and reversible inhibitor of interleukin-converting enzyme	Reduced seizure frequency during the 6-week treatment period	Phase IIa randomized, double-blind clinical trial
siRNA [168]	NLRP3 silencing; caspase-1 silencing	Improved neuronal survival; mitigated seizure severity	Amygdala kindling-induced status epilepticus in a rat model
Amentoflavone [125]	Bioflavonoid with anti-inflammatory effect	Decreased seizure intensity and cognitive impairment by inhibiting the NLRP3 inflammasome	Intraperitoneally injected with PTZ in C57BL/6 mouse model
Melatonin and Sodium valproate [201]	Anti-convulsivant effect	Increased latency; decreased severity of audiogenic seizures	Daily acoustic stimulation for 20 days until the development of persistent myoclonic seizures in a rat model
Curcumin [179]	NLRP3 inhibitor	Improvement of recognition deficiency; reduced hippocampal expression of NLRP3 inflammasome components	Intraperitoneally injection of KA in Sprague Dawley rats
CY-09 [203]	NLRP3 inhibitor	Improved PTZ-induced neuronal loss; lessened astrocyte activation; reduced IL-1β and IL-18 secretion	PTZ-induced mice model
MCC950 [204]	NLRP3 inhibitor	Significantly ameliorated neuronal loss	in vitro and in vivo epileptic models

### Structure–Activity Relationship for Therapeutic Compound Prediction

Therapeutic compound prediction based on structure–activity relationship (SAR) is a crucial aspect of drug discovery and development, particularly in the context of neurological disorders like epilepsy [206]. The process of SAR begins with the identification of potential drug targets related to the pathophysiology of epilepsy. These targets may include ion channels, receptors, or enzymes involved in the regulation of neuronal excitability. With this technique, it is possible to explore the chemical space around these targets, designing and synthesizing compounds with variations in their chemical structures. High-throughput screening methods assess the biological activity of these molecules, providing data on their efficacy and potential side effects. This data is then correlated with the chemical features of the compounds, forming the basis for SAR analysis.

Historically, epilepsy has been managed with a spectrum of antiepileptic drugs (AEDs), each with its unique chemical structure and mode of action. Accordingly, several studies about the SAR of these compounds in both in vitro and in vivo epileptic models are reported ([207,208,209]). However, clinical trials mark a critical juncture where compounds move from promising preclinical findings to large-scale human testing, determining their efficacy and safety in real-world scenarios. Carisbamate is a felbamate analogue with a monocarbamate moiety, differing from felbamate’s dicarbamate structure [210]. Due to this specific structure, carisbamate functions by inhibiting voltage-gated sodium channels (VGSCs) and has shown efficacy in various epilepsy models, indicating a broad spectrum of anticonvulsant properties. Carisbamate is currently in Phase III clinical trials [211] for both monotherapy and adjunctive therapy in treating epilepsy, highlighting its potential as a promising therapeutic option in the management of seizures [212,213]. Another anticonvulsant compound involved in phase III clinical trials is brivaracetam [214]. Interestingly, preliminary SAR studies reported that a benzyl substituent in the amide group and benzhydrylpiperazinyl is crucial for the activity [215]. Among specific NLRP3 inhibitors, several SAR studies in vitro and in vivo highlighted their action mechanism. Accordingly, it has been reported that MCC950 interacts with the Walker B motif within the NLRP3 NACHT domain, preventing NLRP3-mediated ATP to ADP hydrolysis [198,216,217]. Moreover, CY-09 acts upstream of ASC oligomerization to inhibit the subsequent caspase-1 activation and IL-1β production, directly binding to NLRP3 and inhibiting its ATPase activity [218]. In an even more recent paper analyzing the role of curcumin analogues on seizure models, interesting pharmacophoric information about the anti-seizure activity of curcumin analogues is provided [219]. Unfortunately, even if these compounds are tested in pre-clinical studies, to the author’s knowledge, no evidence is reported in the literature about epileptic clinical studies involving these compounds. The importance of the SAR studies necessary to identify functional, structural components together with the gaps present in the literature regarding the use of specific NLRP3 inhibitors in the clinical field further highlight the need to delve deeper into these research topics in order to obtain drugs with a mechanism of action increasingly selective and performing.

## 4. Conclusions

The intricate neuroinflammatory response orchestrates a delicate balance between protective and detrimental effects within the central nervous system. Acute inflammation, characterized by a rapid and controlled response, plays a crucial role in neutralizing danger signals and initiating regenerative processes. However, persistent and uncontrolled inflammation, often driven by chronic activation of glial cells and the NLRP3 inflammasome, can lead to neurodegeneration and contribute to the progression of various CNS disorders. Given the critical importance of this process, it is crucial to understand the intricate mechanisms underlying neuroinflammation and the pivotal role of glial cells and the NLRP3 inflammasome in maintaining CNS homeostasis. By comprehending these mechanisms, it is possible to identify therapeutic strategies aimed at mitigating the detrimental effects of chronic inflammation and promoting neuroprotection, thereby offering new avenues for treating neurodegenerative diseases and enhancing overall brain health. The correlation between NLRP3 levels and the severity of seizures highlights the potential diagnostic and prognostic value of these markers in clinical settings, shedding light on future therapeutic strategies aimed at mitigating the detrimental effects of neuroinflammation in epilepsy.

## Data Availability

Not applicable.

## Abbreviations

AD	Alzheimer’s disease
AIM2	absent in melanoma 2
ASC	apoptosis-associated speck-like protein containing a caspase recruitment domain (CARD)
BBB	blood–brain barrier
BDNF	brain-derived neurotrophic factor
CA	Ammon’s horn
CARD	caspase recruitment domain
CCL	chemokine (C-C motif) ligand
Cl^−^	chloride
CLICs	chloride intracellular channel
CNS	central nervous system
CXCL	chemokine (C-X-C motif) ligand
DAMPs	damage-associated molecular patterns
DG	dentate gyrus
EAE	experimental autoimmune encephalomyelitis
FADD	FAS-associated death domain protein
GENT	gentiopicroside
GFAP	glial fibrillary acidic protein
GPR	120 G protein-coupled receptor 120
GSDMD	gasdermin-D
hPSC	human pluripotent stem cells
HS	hippocampal sclerosis
i.p.	intraperitoneal
ICR	Institute of Cancer Research
IFN-γ	interferon-γ
IL-1	interleukin-1
IL-18	interleukin-18
IL-1β	interleukin-1 beta
IL-6	interleukin-6
iPSC	induced pluripotent stem cells
K^+^	potassium
KA	kainic acid
KO	knockout
LPS	lypopolisaccharide
LRR	leucine-rich repeat
MCU	mitochondrial calcium uniporter
MFS	mossy fiber sprouting
mTLE	mesial temporal lobe epilepsy
NEK7	NIMA-related kinase 7
NF-κB	nuclear factor-kappa B
NLR	nucleotide-binding oligomerization domain (NOD), leucine-rich repeat (LRR)-containing protein receptor (NLR)
NLRP3	nucleotide-binding oligomerization domain (NOD)-like receptor pyrin domain containing 3
NO	nitric oxide
NOD	nucleotide-binding oligomerization domain
OHSC	organotypic hippocampal slice cultures
PAMPs	pathogen-associated molecular patterns
PD	Parkinson’s disease
PML	promyelocytic leukemia protein
PRRs	pattern recognition receptors
PTX	picrotoxin
PTZ	pentylenetetrazole
PYD	pyrin domain
RLD	repeated low doses
ROS	reactive oxygen species
SE	status epilepticus
SRSs	spontaneous recurrent seizures
Stat3	signal transduction activator of transcription 3
TBI	traumatic brain injury
TGF-β	transforming growth factor-beta
TLRs	toll-like receptors
TNF	tumor necrosis factor
TRPM2	transient receptor potential melastatin 2
VEGF	vasoactive endothelial growth factor
WHO	World Health organization
WT	wild type

## References

[1] Roh J.S., Sohn D.H. (2018). Damage-Associated Molecular Patterns in Inflammatory Diseases. Immune Netw..

[2] Piccinini A.M., Midwood K.S. (2010). DAMPening Inflammation by Modulating TLR Signalling. Mediat. Inflamm..

[3] Newton K., Dixit V.M. (2012). Signaling in Innate Immunity and Inflammation. Cold Spring Harb. Perspect. Biol..

[4] Mogensen T.H. (2009). Pathogen Recognition and Inflammatory Signaling in Innate Immune Defenses. Clin. Microbiol. Rev..

[5] Kawai T., Akira S. (2010). The Role of Pattern-Recognition Receptors in Innate Immunity: Update on Toll-like Receptors. Nat. Immunol..

[6] Shabab T., Khanabdali R., Moghadamtousi S.Z., Kadir H.A., Mohan G. (2017). Neuroinflammation Pathways: A General Review. Int. J. Neurosci..

[7] Allen N.J., Lyons D.A. (2018). Glia as Architects of Central Nervous System Formation and Function. Science.

[8] Abbott N.J., Rönnbäck L., Hansson E. (2006). Astrocyte-Endothelial Interactions at the Blood-Brain Barrier. Nat. Rev. Neurosci..

[9] Cabezas R., Avila M., Gonzalez J., El-Bachá R.S., Báez E., García-Segura L.M., Jurado Coronel J.C., Capani F., Cardona-Gomez G.P., Barreto G.E. (2014). Astrocytic Modulation of Blood Brain Barrier: Perspectives on Parkinson’s Disease. Front. Cell. Neurosci.

[10] Koehler R.C., Gebremedhin D., Harder D.R. (2006). Role of Astrocytes in Cerebrovascular Regulation. J. Appl. Physiol..

[11] Heithoff B.P., George K.K., Phares A.N., Zuidhoek I.A., Munoz-Ballester C., Robel S. (2021). Astrocytes Are Necessary for Blood-Brain Barrier Maintenance in the Adult Mouse Brain. Glia.

[12] Chung W.-S., Allen N.J., Eroglu C. (2015). Astrocytes Control Synapse Formation, Function, and Elimination. Cold Spring Harb. Perspect. Biol..

[13] Philips T., Rothstein J.D. (2017). Oligodendroglia: Metabolic Supporters of Neurons. J. Clin. Investig..

[14] Stadelmann C., Timmler S., Barrantes-Freer A., Simons M. (2019). Myelin in the Central Nervous System: Structure, Function, and Pathology. Physiol. Rev..

[15] Norris G.T., Kipnis J. (2019). Immune Cells and CNS Physiology: Microglia and Beyond. J. Exp. Med..

[16] Thion M.S., Ginhoux F., Garel S. (2018). Microglia and Early Brain Development: An Intimate Journey. Science.

[17] Parkhurst C.N., Yang G., Ninan I., Savas J.N., Yates J.R., Lafaille J.J., Hempstead B.L., Littman D.R., Gan W.-B. (2013). Microglia Promote Learning-Dependent Synapse Formation through Brain-Derived Neurotrophic Factor. Cell.

[18] Linnerbauer M., Wheeler M.A., Quintana F.J. (2020). Astrocyte Crosstalk in CNS Inflammation. Neuron.

[19] Voskuhl R.R., Peterson R.S., Song B., Ao Y., Morales L.B.J., Tiwari-Woodruff S., Sofroniew M.V. (2009). Reactive Astrocytes Form Scar-like Perivascular Barriers to Leukocytes during Adaptive Immune Inflammation of the CNS. J. Neurosci..

[20] Olson J.K., Miller S.D. (2004). Microglia Initiate Central Nervous System Innate and Adaptive Immune Responses through Multiple TLRs. J. Immunol..

[21] Bsibsi M., Ravid R., Gveric D., van Noort J.M. (2002). Broad Expression of Toll-like Receptors in the Human Central Nervous System. J. Neuropathol. Exp. Neurol..

[22] Li L., Acioglu C., Heary R.F., Elkabes S. (2021). Role of Astroglial Toll-like Receptors (TLRs) in Central Nervous System Infections, Injury and Neurodegenerative Diseases. Brain Behav. Immun..

[23] Escartin C., Galea E., Lakatos A., O’Callaghan J.P., Petzold G.C., Serrano-Pozo A., Steinhäuser C., Volterra A., Carmignoto G., Agarwal A. (2021). Reactive Astrocyte Nomenclature, Definitions, and Future Directions. Nat. Neurosci..

[24] Escartin C., Guillemaud O., Carrillo-de Sauvage M.-A. (2019). Questions and (Some) Answers on Reactive Astrocytes. Glia.

[25] Pekny M., Johansson C.B., Eliasson C., Stakeberg J., Wallén A., Perlmann T., Lendahl U., Betsholtz C., Berthold C.H., Frisén J. (1999). Abnormal Reaction to Central Nervous System Injury in Mice Lacking Glial Fibrillary Acidic Protein and Vimentin. J. Cell Biol..

[26] Linnerbauer M., Rothhammer V. (2020). Protective Functions of Reactive Astrocytes Following Central Nervous System Insult. Front. Immunol.

[27] Cédile O., Wlodarczyk A., Owens T. (2017). CCL 2 Recruits T Cells into the Brain in a CCR 2-Independent Manner. APMIS.

[28] Amatruda M., Chapouly C., Woo V., Safavi F., Zhang J., Dai D., Therattil A., Moon C., Villavicencio J., Gordon A. (2022). Astrocytic Junctional Adhesion Molecule-A Regulates T-Cell Entry Past the Glia Limitans to Promote Central Nervous System Autoimmune Attack. Brain Commun..

[29] Gajtkó A., Bakk E., Hegedűs K., Ducza L., Holló K. (2020). IL-1β Induced Cytokine Expression by Spinal Astrocytes Can Play a Role in the Maintenance of Chronic Inflammatory Pain. Front. Physiol..

[30] Gimenez M.A., Sim J., Archambault A.S., Klein R.S., Russell J.H. (2006). A Tumor Necrosis Factor Receptor 1-Dependent Conversation between Central Nervous System-Specific T Cells and the Central Nervous System Is Required for Inflammatory Infiltration of the Spinal Cord. Am. J. Pathol..

[31] Papazian I., Tsoukala E., Boutou A., Karamita M., Kambas K., Iliopoulou L., Fischer R., Kontermann R.E., Denis M.C., Kollias G. (2021). Fundamentally Different Roles of Neuronal TNF Receptors in CNS Pathology: TNFR1 and IKKβ Promote Microglial Responses and Tissue Injury in Demyelination While TNFR2 Protects against Excitotoxicity in Mice. J. Neuroinflammation.

[32] Argaw A.T., Asp L., Zhang J., Navrazhina K., Pham T., Mariani J.N., Mahase S., Dutta D.J., Seto J., Kramer E.G. (2012). Astrocyte-Derived VEGF-A Drives Blood-Brain Barrier Disruption in CNS Inflammatory Disease. J. Clin. Investig..

[33] Cekanaviciute E., Dietrich H.K., Axtell R.C., Williams A.M., Egusquiza R., Wai K.M., Koshy A.A., Buckwalter M.S. (2014). Astrocytic TGF-β Signaling Limits Inflammation and Reduces Neuronal Damage during Central Nervous System Toxoplasma Infection. J. Immunol..

[34] Cekanaviciute E., Fathali N., Doyle K.P., Williams A.M., Han J., Buckwalter M.S. (2014). Astrocytic Transforming Growth Factor-Beta Signaling Reduces Subacute Neuroinflammation after Stroke in Mice. Glia.

[35] Woodburn S.C., Bollinger J.L., Wohleb E.S. (2021). The Semantics of Microglia Activation: Neuroinflammation, Homeostasis, and Stress. J. Neuroinflammation.

[36] Anttila J.E., Whitaker K.W., Wires E.S., Harvey B.K., Airavaara M. (2017). Role of Microglia in Ischemic Focal Stroke and Recovery: Focus on Toll-like Receptors. Prog. Neuro-Psychopharmacol. Biol. Psychiatry.

[37] Heindl S., Gesierich B., Benakis C., Llovera G., Duering M., Liesz A. (2018). Automated Morphological Analysis of Microglia After Stroke. Front. Cell. Neurosci..

[38] Lynch M.A. (2009). The Multifaceted Profile of Activated Microglia. Mol. Neurobiol..

[39] Smith J.A., Das A., Ray S.K., Banik N.L. (2012). Role of Pro-Inflammatory Cytokines Released from Microglia in Neurodegenerative Diseases. Brain Res. Bull..

[40] Liu T., Zhang L., Joo D., Sun S.-C. (2017). NF-ΚB Signaling in Inflammation. Signal Transduct. Target. Ther..

[41] Sutterwala F.S., Haasken S., Cassel S.L. (2014). Mechanism of NLRP3 Inflammasome Activation. Ann. N. Y. Acad. Sci..

[42] Li R., Xu W., Chen Y., Qiu W., Shu Y., Wu A., Dai Y., Bao J., Lu Z., Hu X. (2014). Raloxifene Suppresses Experimental Autoimmune Encephalomyelitis and NF-ΚB-Dependent CCL20 Expression in Reactive Astrocytes. PLoS ONE.

[43] Liddelow S.A., Guttenplan K.A., Clarke L.E., Bennett F.C., Bohlen C.J., Schirmer L., Bennett M.L., Münch A.E., Chung W.-S., Peterson T.C. (2017). Neurotoxic Reactive Astrocytes are Induced by Activated Microglia. Nature.

[44] Olude M.A., Mouihate A., Mustapha O.A., Farina C., Quintana F.J., Olopade J.O. (2022). Astrocytes and Microglia in Stress-Induced Neuroinflammation: The African Perspective. Front. Immunol..

[45] Rodríguez A.M., Rodríguez J., Giambartolomei G.H. (2022). Microglia at the Crossroads of Pathogen-Induced Neuroinflammation. ASN Neuro.

[46] Cherry J.D., Olschowka J.A., O’Banion M.K. (2014). Neuroinflammation and M2 Microglia: The Good, the Bad, and the Inflamed. J. Neuroinflammation.

[47] Broz P., Dixit V.M. (2016). Inflammasomes: Mechanism of Assembly, Regulation and Signalling. Nat. Rev. Immunol..

[48] Lu A., Wu H. (2015). Structural Mechanisms of Inflammasome Assembly. FEBS J.

[49] Sharma D., Kanneganti T.-D. (2016). The Cell Biology of Inflammasomes: Mechanisms of Inflammasome Activation and Regulation. J. Cell Biol..

[50] Guo H., Callaway J.B., Ting J.P.-Y. (2015). Inflammasomes: Mechanism of Action, Role in Disease, and Therapeutics. Nat. Med..

[51] Bergsbaken T., Fink S.L., Cookson B.T. (2009). Pyroptosis: Host Cell Death and Inflammation. Nat. Rev. Microbiol..

[52] Missiroli S., Perrone M., Boncompagni C., Borghi C., Campagnaro A., Marchetti F., Anania G., Greco P., Fiorica F., Pinton P. (2021). Targeting the NLRP3 Inflammasome as a New Therapeutic Option for Overcoming Cancer. Cancers.

[53] Vezzani B., Carinci M., Patergnani S., Pasquin M.P., Guarino A., Aziz N., Pinton P., Simonato M., Giorgi C. (2020). The Dichotomous Role of Inflammation in the CNS: A Mitochondrial Point of View. Biomolecules.

[54] Carinci M., Vezzani B., Patergnani S., Ludewig P., Lessmann K., Magnus T., Casetta I., Pugliatti M., Pinton P., Giorgi C. (2021). Different Roles of Mitochondria in Cell Death and Inflammation: Focusing on Mitochondrial Quality Control in Ischemic Stroke and Reperfusion. Biomedicines.

[55] Missiroli S., Genovese I., Perrone M., Vezzani B., Vitto V.A.M., Giorgi C. (2020). The Role of Mitochondria in Inflammation: From Cancer to Neurodegenerative Disorders. J. Clin. Med..

[56] Bouhamida E., Morciano G., Perrone M., Kahsay A.E., Della Sala M., Wieckowski M.R., Fiorica F., Pinton P., Giorgi C., Patergnani S. (2022). The Interplay of Hypoxia Signaling on Mitochondrial Dysfunction and Inflammation in Cardiovascular Diseases and Cancer: From Molecular Mechanisms to Therapeutic Approaches. Biology.

[57] Ohto U., Kamitsukasa Y., Ishida H., Zhang Z., Murakami K., Hirama C., Maekawa S., Shimizu T. (2022). Structural Basis for the Oligomerization-Mediated Regulation of NLRP3 Inflammasome Activation. Proc. Natl. Acad. Sci. USA.

[58] Swanson K.V., Deng M., Ting J.P.-Y. (2019). The NLRP3 Inflammasome: Molecular Activation and Regulation to Therapeutics. Nat. Rev. Immunol/.

[59] Kelley N., Jeltema D., Duan Y., He Y. (2019). The NLRP3 Inflammasome: An Overview of Mechanisms of Activation and Regulation. Int. J. Mol. Sci/.

[60] Huang Y., Xu W., Zhou R. (2021). NLRP3 Inflammasome Activation and Cell Death. Cell. Mol. Immunol..

[61] Gurung P., Anand P.K., Malireddi R.K.S., Vande Walle L., Van Opdenbosch N., Dillon C.P., Weinlich R., Green D.R., Lamkanfi M., Kanneganti T.-D. (2014). FADD and Caspase-8 Mediate Priming and Activation of the Canonical and Noncanonical Nlrp3 Inflammasomes. J. Immunol..

[62] Lemmers B., Salmena L., Bidère N., Su H., Matysiak-Zablocki E., Murakami K., Ohashi P.S., Jurisicova A., Lenardo M., Hakem R. (2007). Essential Role for Caspase-8 in Toll-like Receptors and NFkappaB Signaling. J. Biol. Chem..

[63] Lin K.-M., Hu W., Troutman T.D., Jennings M., Brewer T., Li X., Nanda S., Cohen P., Thomas J.A., Pasare C. (2014). IRAK-1 Bypasses Priming and Directly Links TLRs to Rapid NLRP3 Inflammasome Activation. Proc. Natl. Acad. Sci. USA.

[64] Juliana C., Fernandes-Alnemri T., Kang S., Farias A., Qin F., Alnemri E.S. (2012). Non-Transcriptional Priming and Deubiquitination Regulate NLRP3 Inflammasome Activation. J. Biol. Chem..

[65] Yang Y., Wang H., Kouadir M., Song H., Shi F. (2019). Recent Advances in the Mechanisms of NLRP3 Inflammasome Activation and Its Inhibitors. Cell Death Dis..

[66] McKee C.M., Coll R.C. (2020). NLRP3 Inflammasome Priming: A Riddle Wrapped in a Mystery inside an Enigma. J. Leukoc. Biol..

[67] Muñoz-Planillo R., Kuffa P., Martínez-Colón G., Smith B.L., Rajendiran T.M., Núñez G. (2013). K^+^ Efflux Is the Common Trigger of NLRP3 Inflammasome Activation by Bacterial Toxins and Particulate Matter. Immunity.

[68] Karmakar M., Katsnelson M.A., Dubyak G.R., Pearlman E. (2016). Neutrophil P2X7 Receptors Mediate NLRP3 Inflammasome-Dependent IL-1β Secretion in Response to ATP. Nat. Commun..

[69] Di Virgilio F., Dal Ben D., Sarti A.C., Giuliani A.L., Falzoni S. (2017). The P2X7 Receptor in Infection and Inflammation. Immunity.

[70] Tang T., Lang X., Xu C., Wang X., Gong T., Yang Y., Cui J., Bai L., Wang J., Jiang W. (2017). CLICs-Dependent Chloride Efflux Is an Essential and Proximal Upstream Event for NLRP3 Inflammasome Activation. Nat. Commun..

[71] Domingo-Fernández R., Coll R.C., Kearney J., Breit S., O’Neill L.A.J. (2017). The Intracellular Chloride Channel Proteins CLIC1 and CLIC4 Induce IL-1β Transcription and Activate the NLRP3 Inflammasome. J. Biol. Chem..

[72] He Y., Zeng M.Y., Yang D., Motro B., Núñez G. (2016). NEK7 Is an Essential Mediator of NLRP3 Activation Downstream of Potassium Efflux. Nature.

[73] Chen X., Liu G., Yuan Y., Wu G., Wang S., Yuan L. (2019). NEK7 Interacts with NLRP3 to Modulate the Pyroptosis in Inflammatory Bowel Disease via NF-ΚB Signaling. Cell Death Dis..

[74] Green J.P., Yu S., Martín-Sánchez F., Pelegrin P., Lopez-Castejon G., Lawrence C.B., Brough D. (2018). Chloride Regulates Dynamic NLRP3-Dependent ASC Oligomerization and Inflammasome Priming. Proc. Natl. Acad. Sci. USA.

[75] Lee G.-S., Subramanian N., Kim A.I., Aksentijevich I., Goldbach-Mansky R., Sacks D.B., Germain R.N., Kastner D.L., Chae J.J. (2012). The Calcium-Sensing Receptor Regulates the NLRP3 Inflammasome through Ca^2+^ and CAMP. Nature.

[76] Murakami T., Ockinger J., Yu J., Byles V., McColl A., Hofer A.M., Horng T. (2012). Critical Role for Calcium Mobilization in Activation of the NLRP3 Inflammasome. Proc. Natl. Acad. Sci. USA.

[77] Giorgi C., Danese A., Missiroli S., Patergnani S., Pinton P. (2018). Calcium Dynamics as a Machine for Decoding Signals. Trends Cell Biol..

[78] Di A., Xiong S., Ye Z., Malireddi R.K.S., Kometani S., Zhong M., Mittal M., Hong Z., Kanneganti T.-D., Rehman J. (2018). The TWIK2 Potassium Efflux Channel in Macrophages Mediates NLRP3 Inflammasome-Induced Inflammation. Immunity.

[79] Shi J., Zhao Y., Wang K., Shi X., Wang Y., Huang H., Zhuang Y., Cai T., Wang F., Shao F. (2015). Cleavage of GSDMD by Inflammatory Caspases Determines Pyroptotic Cell Death. Nature.

[80] He W., Wan H., Hu L., Chen P., Wang X., Huang Z., Yang Z.-H., Zhong C.-Q., Han J. (2015). Gasdermin D Is an Executor of Pyroptosis and Required for Interleukin-1β Secretion. Cell Res..

[81] Russo M.V., McGavern D.B. (2016). Inflammatory Neuroprotection Following Traumatic Brain Injury. Science.

[82] Walker K.A. (2018). Inflammation and Neurodegeneration: Chronicity Matters. Aging.

[83] Ransohoff R.M. (2016). How Neuroinflammation Contributes to Neurodegeneration. Science.

[84] Tang Y., Le W. (2016). Differential Roles of M1 and M2 Microglia in Neurodegenerative Diseases. Mol. Neurobiol..

[85] Simpson D.S.A., Oliver P.L. (2020). ROS Generation in Microglia: Understanding Oxidative Stress and Inflammation in Neurodegenerative Disease. Antioxidants.

[86] Umpierre A.D., Wu L.-J. (2021). How Microglia Sense and Regulate Neuronal Activity. Glia.

[87] Russo M.V., McGavern D.B. (2015). Immune Surveillance of the CNS Following Infection and Injury. Trends. Immunol..

[88] Rothhammer V., Quintana F.J. (2015). Control of Autoimmune CNS Inflammation by Astrocytes. Semin. Immunopathol..

[89] John G.R., Chen L., Rivieccio M.A., Melendez-Vasquez C.V., Hartley A., Brosnan C.F. (2004). Interleukin-1beta Induces a Reactive Astroglial Phenotype via Deactivation of the Rho GTPase-Rock Axis. J. Neurosci..

[90] Saha R.N., Liu X., Pahan K. (2006). Up-Regulation of BDNF in Astrocytes by TNF-Alpha: A Case for the Neuroprotective Role of Cytokine. J. Neuroimmune Pharmacol..

[91] Guan Y., Han F. (2020). Key Mechanisms and Potential Targets of the NLRP3 Inflammasome in Neurodegenerative Diseases. Front. Integr. Neurosci..

[92] Holbrook J.A., Jarosz-Griffiths H.H., Caseley E., Lara-Reyna S., Poulter J.A., Williams-Gray C.H., Peckham D., McDermott M.F. (2021). Neurodegenerative Disease and the NLRP3 Inflammasome. Front. Pharmacol..

[93] Saresella M., La Rosa F., Piancone F., Zoppis M., Marventano I., Calabrese E., Rainone V., Nemni R., Mancuso R., Clerici M. (2016). The NLRP3 and NLRP1 Inflammasomes Are Activated in Alzheimer’s Disease. Mol. Neurodegener..

[94] Stancu I.-C., Cremers N., Vanrusselt H., Couturier J., Vanoosthuyse A., Kessels S., Lodder C., Brône B., Huaux F., Octave J.-N. (2019). Aggregated Tau Activates NLRP3-ASC Inflammasome Exacerbating Exogenously Seeded and Non-Exogenously Seeded Tau Pathology in Vivo. Acta Neuropathol..

[95] von Herrmann K.M., Salas L.A., Martinez E.M., Young A.L., Howard J.M., Feldman M.S., Christensen B.C., Wilkins O.M., Lee S.L., Hickey W.F. (2018). NLRP3 Expression in Mesencephalic Neurons and Characterization of a Rare NLRP3 Polymorphism Associated with Decreased Risk of Parkinson’s Disease. NPJ Parkinson’s Dis..

[96] Fan Z., Pan Y.-T., Zhang Z.-Y., Yang H., Yu S.-Y., Zheng Y., Ma J.-H., Wang X.-M. (2020). Systemic Activation of NLRP3 Inflammasome and Plasma α-Synuclein Levels Are Correlated with Motor Severity and Progression in Parkinson’s Disease. J. Neuroinflammation.

[97] Zhang P., Shao X.-Y., Qi G.-J., Chen Q., Bu L.-L., Chen L.-J., Shi J., Ming J., Tian B. (2016). Cdk5-Dependent Activation of Neuronal Inflammasomes in Parkinson’s Disease. Mov. Disord..

[98] Barclay W., Shinohara M.L. (2017). Inflammasome Activation in Multiple Sclerosis and Experimental Autoimmune Encephalomyelitis (EAE). Brain Pathol..

[99] Gris D., Ye Z., Iocca H.A., Wen H., Craven R.R., Gris P., Huang M., Schneider M., Miller S.D., Ting J.P.-Y. (2010). NLRP3 Plays a Critical Role in the Development of Experimental Autoimmune Encephalomyelitis by Mediating Th1 and Th17 Responses. J. Immunol..

[100] Beghi E. (2020). The Epidemiology of Epilepsy. Neuroepidemiology.

[101] Panayiotopoulos C.P. (2005). The Epilepsies: Seizures, Syndromes and Management.

[102] Wang J., Lin Z.-J., Liu L., Xu H.-Q., Shi Y.-W., Yi Y.-H., He N., Liao W.-P. (2017). Epilepsy-Associated Genes. Seizure.

[103] Pitkänen A., Lukasiuk K., Dudek F.E., Staley K.J. (2015). Epileptogenesis. Cold Spring Harb. Perspect. Med..

[104] Klein P., Dingledine R., Aronica E., Bernard C., Blümcke I., Boison D., Brodie M.J., Brooks-Kayal A.R., Engel J., Forcelli P.A. (2018). Commonalities in Epileptogenic Processes from Different Acute Brain Insults: Do They Translate?. Epilepsia.

[105] Pitkänen A., Lukasiuk K. (2011). Mechanisms of Epileptogenesis and Potential Treatment Targets. Lancet Neurol..

[106] Devinsky O., Vezzani A., O’Brien T.J., Jette N., Scheffer I.E., de Curtis M., Perucca P. (2018). Epilepsy. Nat. Rev. Dis. Primers.

[107] Kanner A.M., Mazarati A., Koepp M. (2014). Biomarkers of Epileptogenesis: Psychiatric Comorbidities (?). Neurotherapeutics.

[108] Blümcke I., Thom M., Aronica E., Armstrong D.D., Bartolomei F., Bernasconi A., Bernasconi N., Bien C.G., Cendes F., Coras R. (2013). International Consensus Classification of Hippocampal Sclerosis in Temporal Lobe Epilepsy: A Task Force Report from the ILAE Commission on Diagnostic Methods. Epilepsia.

[109] Desmedt J.E., Borenstein S. (1973). Collateral Innervation of Muscle Fibres by Motor Axons of Dystrophic Motor Units. Nature.

[110] Paradiso B., Marconi P., Zucchini S., Berto E., Binaschi A., Bozac A., Buzzi A., Mazzuferi M., Magri E., Navarro Mora G. (2009). Localized Delivery of Fibroblast Growth Factor-2 and Brain-Derived Neurotrophic Factor Reduces Spontaneous Seizures in an Epilepsy Model. Proc. Natl. Acad. Sci. USA.

[111] Cross D.J., Cavazos J.E. (2009). PLASTICITY|Circuitry Reorganization, Regeneration, and Sprouting. Encyclopedia of Basic Epilepsy Research.

[112] Paradiso B., Zucchini S., Su T., Bovolenta R., Berto E., Marconi P., Marzola A., Navarro Mora G., Fabene P.F., Simonato M. (2011). Localized Overexpression of FGF-2 and BDNF in Hippocampus Reduces Mossy Fiber Sprouting and Spontaneous Seizures up to 4 Weeks after Pilocarpine-Induced Status Epilepticus. Epilepsia.

[113] Houser C.R., Miyashiro J.E., Swartz B.E., Walsh G.O., Rich J.R., Delgado-Escueta A.V. (1990). Altered Patterns of Dynorphin Immunoreactivity Suggest Mossy Fiber Reorganization in Human Hippocampal Epilepsy. J. Neurosci..

[114] Kitaura H., Shirozu H., Masuda H., Fukuda M., Fujii Y., Kakita A. (2018). Pathophysiological Characteristics Associated With Epileptogenesis in Human Hippocampal Sclerosis. EBioMedicine.

[115] Buckmaster P.S., Zhang G.F., Yamawaki R. (2002). Axon Sprouting in a Model of Temporal Lobe Epilepsy Creates a Predominantly Excitatory Feedback Circuit. J. Neurosci..

[116] Scharfman H.E., Sollas A.L., Berger R.E., Goodman J.H. (2003). Electrophysiological Evidence of Monosynaptic Excitatory Transmission between Granule Cells after Seizure-Induced Mossy Fiber Sprouting. J. Neurophysiol..

[117] Bovolenta R., Zucchini S., Paradiso B., Rodi D., Merigo F., Navarro Mora G., Osculati F., Berto E., Marconi P., Marzola A. (2010). Hippocampal FGF-2 and BDNF Overexpression Attenuates Epileptogenesis-Associated Neuroinflammation and Reduces Spontaneous Recurrent Seizures. J. Neuroinflammation.

[118] Vezzani A., French J., Bartfai T., Baram T.Z. (2011). The Role of Inflammation in Epilepsy. Nat. Rev. Neurol..

[119] Giordano G., Costa L.G. (2011). Primary Neurons in Culture and Neuronal Cell Lines for in Vitro Neurotoxicological Studies. Methods Mol. Biol..

[120] Kiese K., Jablonski J., Hackenbracht J., Wrosch J.K., Groemer T.W., Kornhuber J., Blümcke I., Kobow K. (2017). Epigenetic Control of Epilepsy Target Genes Contributes to a Cellular Memory of Epileptogenesis in Cultured Rat Hippocampal Neurons. Acta Neuropathol. Commun..

[121] Costamagna G., Andreoli L., Corti S., Faravelli I. (2019). IPSCs-Based Neural 3D Systems: A Multidimensional Approach for Disease Modeling and Drug Discovery. Cells.

[122] Magalhães D.M., Pereira N., Rombo D.M., Beltrão-Cavacas C., Sebastião A.M., Valente C.A. (2018). Ex Vivo Model of Epilepsy in Organotypic Slices-a New Tool for Drug Screening. J. Neuroinflammation.

[123] Valente C.A., Meda F.J., Carvalho M., Sebastião A.M. (2021). A Model of Epileptogenesis in Rhinal Cortex-Hippocampus Organotypic Slice Cultures. J. Vis. Exp..

[124] Jablonski J., Hoffmann L., Blümcke I., Fejtová A., Uebe S., Ekici A.B., Gnatkovsky V., Kobow K. (2021). Experimental Epileptogenesis in a Cell Culture Model of Primary Neurons from Rat Brain: A Temporal Multi-Scale Study. Cells.

[125] Rong S., Wan D., Fan Y., Liu S., Sun K., Huo J., Zhang P., Li X., Xie X., Wang F. (2019). Amentoflavone Affects Epileptogenesis and Exerts Neuroprotective Effects by Inhibiting NLRP3 Inflammasome. Front. Pharmacol..

[126] Sun Y., Ma J., Li D., Li P., Zhou X., Li Y., He Z., Qin L., Liang L., Luo X. (2019). Interleukin-10 Inhibits Interleukin-1β Production and Inflammasome Activation of Microglia in Epileptic Seizures. J. Neuroinflammation.

[127] Zhang H., Yu S., Xia L., Peng X., Wang S., Yao B. (2022). NLRP3 Inflammasome Activation Enhances ADK Expression to Accelerate Epilepsy in Mice. Neurochem. Res..

[128] Goshi N., Morgan R.K., Lein P.J., Seker E. (2022). Correction to: A Primary Neural Cell Culture Model to Study Neuron, Astrocyte, and Microglia Interactions in Neuroinflammation. J. Neuroinflammation.

[129] Goshi N., Kim H., Girardi G., Gardner A., Seker E. (2023). Electrophysiological Activity of Primary Cortical Neuron-Glia Mixed Cultures. Cells.

[130] Di Lullo E., Kriegstein A.R. (2017). The Use of Brain Organoids to Investigate Neural Development and Disease. Nat. Rev. Neurosci..

[131] Avior Y., Sagi I., Benvenisty N. (2016). Pluripotent Stem Cells in Disease Modelling and Drug Discovery. Nat. Rev. Mol. Cell Biol..

[132] Eiraku M., Watanabe K., Matsuo-Takasaki M., Kawada M., Yonemura S., Matsumura M., Wataya T., Nishiyama A., Muguruma K., Sasai Y. (2008). Self-Organized Formation of Polarized Cortical Tissues from ESCs and Its Active Manipulation by Extrinsic Signals. Cell Stem Cell.

[133] Lancaster M.A., Renner M., Martin C.-A., Wenzel D., Bicknell L.S., Hurles M.E., Homfray T., Penninger J.M., Jackson A.P., Knoblich J.A. (2013). Cerebral Organoids Model Human Brain Development and Microcephaly. Nature.

[134] Lancaster M.A., Knoblich J.A. (2014). Generation of Cerebral Organoids from Human Pluripotent Stem Cells. Nat. Protoc..

[135] Sakaguchi H., Kadoshima T., Soen M., Narii N., Ishida Y., Ohgushi M., Takahashi J., Eiraku M., Sasai Y. (2015). Generation of Functional Hippocampal Neurons from Self-Organizing Human Embryonic Stem Cell-Derived Dorsomedial Telencephalic Tissue. Nat. Commun..

[136] Jo J., Xiao Y., Sun A.X., Cukuroglu E., Tran H.-D., Göke J., Tan Z.Y., Saw T.Y., Tan C.-P., Lokman H. (2016). Midbrain-like Organoids from Human Pluripotent Stem Cells Contain Functional Dopaminergic and Neuromelanin-Producing Neurons. Cell Stem Cell.

[137] Monzel A.S., Smits L.M., Hemmer K., Hachi S., Moreno E.L., van Wuellen T., Jarazo J., Walter J., Brüggemann I., Boussaad I. (2017). Derivation of Human Midbrain-Specific Organoids from Neuroepithelial Stem Cells. Stem Cell Rep..

[138] Qian X., Nguyen H.N., Song M.M., Hadiono C., Ogden S.C., Hammack C., Yao B., Hamersky G.R., Jacob F., Zhong C. (2016). Brain-Region-Specific Organoids Using Mini-Bioreactors for Modeling ZIKV Exposure. Cell.

[139] Muguruma K., Nishiyama A., Kawakami H., Hashimoto K., Sasai Y. (2015). Self-Organization of Polarized Cerebellar Tissue in 3D Culture of Human Pluripotent Stem Cells. Cell Rep..

[140] Ozone C., Suga H., Eiraku M., Kadoshima T., Yonemura S., Takata N., Oiso Y., Tsuji T., Sasai Y. (2016). Functional Anterior Pituitary Generated in Self-Organizing Culture of Human Embryonic Stem Cells. Nat. Commun..

[141] Eiraku M., Takata N., Ishibashi H., Kawada M., Sakakura E., Okuda S., Sekiguchi K., Adachi T., Sasai Y. (2011). Self-Organizing Optic-Cup Morphogenesis in Three-Dimensional Culture. Nature.

[142] Xiang Y., Tanaka Y., Cakir B., Patterson B., Kim K.-Y., Sun P., Kang Y.-J., Zhong M., Liu X., Patra P. (2019). HESC-Derived Thalamic Organoids Form Reciprocal Projections When Fused with Cortical Organoids. Cell Stem Cell.

[143] Sloan S.A., Darmanis S., Huber N., Khan T.A., Birey F., Caneda C., Reimer R., Quake S.R., Barres B.A., Paşca S.P. (2017). Human Astrocyte Maturation Captured in 3D Cerebral Cortical Spheroids Derived from Pluripotent Stem Cells. Neuron.

[144] Dutta D., Heo I., Clevers H. (2017). Disease Modeling in Stem Cell-Derived 3D Organoid Systems. Trends. Mol. Med..

[145] Fatehullah A., Tan S.H., Barker N. (2016). Organoids as an in Vitro Model of Human Development and Disease. Nat. Cell Biol..

[146] Ormel P.R., Vieira de Sá R., van Bodegraven E.J., Karst H., Harschnitz O., Sneeboer M.A.M., Johansen L.E., van Dijk R.E., Scheefhals N., Berdenis van Berlekom A. (2018). Microglia Innately Develop within Cerebral Organoids. Nat. Commun..

[147] Song L., Yuan X., Jones Z., Vied C., Miao Y., Marzano M., Hua T., Sang Q.-X.A., Guan J., Ma T. (2019). Functionalization of Brain Region-Specific Spheroids with Isogenic Microglia-like Cells. Sci. Rep..

[148] Ao Z., Cai H., Wu Z., Song S., Karahan H., Kim B., Lu H.-C., Kim J., Mackie K., Guo F. (2021). Tubular Human Brain Organoids to Model Microglia-Mediated Neuroinflammation. Lab Chip.

[149] Popova G., Soliman S.S., Kim C.N., Keefe M.G., Hennick K.M., Jain S., Li T., Tejera D., Shin D., Chhun B.B. (2021). Human Microglia States are Conserved across Experimental Models and Regulate Neural Stem Cell Responses in Chimeric Organoids. Cell Stem Cell.

[150] Wörsdörfer P., Dalda N., Kern A., Krüger S., Wagner N., Kwok C.K., Henke E., Ergün S. (2019). Generation of Complex Human Organoid Models Including Vascular Networks by Incorporation of Mesodermal Progenitor Cells. Sci. Rep..

[151] Fagerlund I., Dougalis A., Shakirzyanova A., Gómez-Budia M., Pelkonen A., Konttinen H., Ohtonen S., Fazaludeen M.F., Koskuvi M., Kuusisto J. (2021). Microglia-like Cells Promote Neuronal Functions in Cerebral Organoids. Cells.

[152] Sabate-Soler S., Nickels S.L., Saraiva C., Berger E., Dubonyte U., Barmpa K., Lan Y.J., Kouno T., Jarazo J., Robertson G. (2022). Microglia Integration into Human Midbrain Organoids Leads to Increased Neuronal Maturation and Functionality. Glia.

[153] Parent J.M., Anderson S.A. (2015). Reprogramming Patient-Derived Cells to Study the Epilepsies. Nat. Neurosci..

[154] Williams E.C., Zhong X., Mohamed A., Li R., Liu Y., Dong Q., Ananiev G.E., Mok J.C.C., Lin B.R., Lu J. (2014). Mutant Astrocytes Differentiated from Rett Syndrome Patients-Specific IPSCs Have Adverse Effects on Wild-Type Neurons. Hum. Mol. Genet..

[155] Liu Y., Liu H., Sauvey C., Yao L., Zarnowska E.D., Zhang S.-C. (2013). Directed Differentiation of Forebrain GABA Interneurons from Human Pluripotent Stem Cells. Nat. Protoc..

[156] Hoyle C., Redondo-Castro E., Cook J., Tzeng T.-C., Allan S.M., Brough D., Lemarchand E. (2020). Hallmarks of NLRP3 Inflammasome Activation Are Observed in Organotypic Hippocampal Slice Culture. Immunology.

[157] Sundstrom L., Morrison B., Bradley M., Pringle A. (2005). Organotypic Cultures as Tools for Functional Screening in the CNS. Drug. Discov. Today.

[158] Alaylioğlu M., Dursun E., Yilmazer S., Ak D.G. (2020). A Bridge Between in Vitro and in Vivo Studies in Neuroscience: Organotypic Brain Slice Cultures. Noro Psikiyatr. Ars..

[159] Berdichevsky Y., Dzhala V., Mail M., Staley K.J. (2012). Interictal Spikes, Seizures and Ictal Cell Death Are Not Necessary for Post-Traumatic Epileptogenesis in Vitro. Neurobiol. Dis..

[160] Dyhrfjeld-Johnsen J., Berdichevsky Y., Swiercz W., Sabolek H., Staley K.J. (2010). Interictal Spikes Precede Ictal Discharges in an Organotypic Hippocampal Slice Culture Model of Epileptogenesis. J. Clin. Neurophysiol..

[161] Liu J., Saponjian Y., Mahoney M.M., Staley K.J., Berdichevsky Y. (2017). Epileptogenesis in Organotypic Hippocampal Cultures Has Limited Dependence on Culture Medium Composition. PLoS ONE.

[162] Chong S.-A., Balosso S., Vandenplas C., Szczesny G., Hanon E., Claes K., Van Damme X., Danis B., Van Eyll J., Wolff C. (2018). Intrinsic Inflammation Is a Potential Anti-Epileptogenic Target in the Organotypic Hippocampal Slice Model. Neurotherapeutics.

[163] Shaker T., Chattopadhyaya B., Amilhon B., Di Cristo G., Weil A.G. (2021). Transduction of Inflammation from Peripheral Immune Cells to the Hippocampus Induces Neuronal Hyperexcitability Mediated by Caspase-1 Activation. Neurobiol. Dis..

[164] Gong Z., Zhou J., Li H., Gao Y., Xu C., Zhao S., Chen Y., Cai W., Wu J. (2015). Curcumin Suppresses NLRP3 Inflammasome Activation and Protects against LPS-Induced Septic Shock. Mol. Nutr. Food Res..

[165] Drion C.M., Kooijman L., Aronica E., van Vliet E.A., Wadman W.J., Chameau P., Gorter J.A. (2019). Curcumin Reduces Development of Seizurelike Events in the Hippocampal-Entorhinal Cortex Slice Culture Model for Epileptogenesis. Epilepsia.

[166] Toldo S., Mezzaroma E., McGeough M.D., Peña C.A., Marchetti C., Sonnino C., Van Tassell B.W., Salloum F.N., Voelkel N.F., Hoffman H.M. (2015). Independent Roles of the Priming and the Triggering of the NLRP3 Inflammasome in the Heart. Cardiovasc. Res..

[167] Alyu F., Dikmen M. (2017). Inflammatory Aspects of Epileptogenesis: Contribution of Molecular Inflammatory Mechanisms. Acta. Neuropsychiatr..

[168] Meng X.-F., Tan L., Tan M.-S., Jiang T., Tan C.-C., Li M.-M., Wang H.-F., Yu J.-T. (2014). Inhibition of the NLRP3 Inflammasome Provides Neuroprotection in Rats Following Amygdala Kindling-Induced Status Epilepticus. J. Neuroinflammation.

[169] El-Sayed R.M., Fawzy M.N., Zaki H.F., Abd El-Haleim E.A. (2023). Neuroprotection Impact of Biochanin A against Pentylenetetrazol-Kindled Mice: Targeting NLRP3 Inflammasome/TXNIP Pathway and Autophagy Modulation. Int. Immunopharmacol..

[170] Liu R., Wu S., Guo C., Hu Z., Peng J., Guo K., Zhang X., Li J. (2020). Ibuprofen Exerts Antiepileptic and Neuroprotective Effects in the Rat Model of Pentylenetetrazol-Induced Epilepsy via the COX-2/NLRP3/IL-18 Pathway. Neurochem. Res..

[171] Wu C., Zhang G., Chen L., Kim S., Yu J., Hu G., Chen J., Huang Y., Zheng G., Huang S. (2019). The Role of NLRP3 and IL-1β in Refractory Epilepsy Brain Injury. Front. Neurol..

[172] Wang Z., Zhou L., An D., Xu W., Wu C., Sha S., Li Y., Zhu Y., Chen A., Du Y. (2019). TRPV4-Induced Inflammatory Response Is Involved in Neuronal Death in Pilocarpine Model of Temporal Lobe Epilepsy in Mice. Cell Death Dis..

[173] Jiang Q., Tang G., Zhong X.-M., Ding D.-R., Wang H., Li J.-N. (2021). Role of Stat3 in NLRP3/Caspase-1-Mediated Hippocampal Neuronal Pyroptosis in Epileptic Mice. Synapse.

[174] Yue J., He J., Wei Y., Shen K., Wu K., Yang X., Liu S., Zhang C., Yang H. (2020). Decreased Expression of Rev-Erbα in the Epileptic Foci of Temporal Lobe Epilepsy and Activation of Rev-Erbα Have Anti-Inflammatory and Neuroprotective Effects in the Pilocarpine Model. J. Neuroinflammation.

[175] Hu H., Zhu T., Gong L., Zhao Y., Shao Y., Li S., Sun Z., Ling Y., Tao Y., Ying Y. (2020). Transient Receptor Potential Melastatin 2 Contributes to Neuroinflammation and Negatively Regulates Cognitive Outcomes in a Pilocarpine-Induced Mouse Model of Epilepsy. Int. Immunopharmacol.

[176] Zhu J., Park S., Kim C.H., Jeong K.H., Kim W.-J. (2023). Eugenol Alleviates Neuronal Damage via Inhibiting Inflammatory Process against Pilocarpine-Induced Status Epilepticus. Exp. Biol. Med..

[177] Yang W., Ma L., Xu S., Zheng P., Du J., Wu J., Yu J., Sun T. (2023). Gentiopicroside Alleviated Epileptogenesis in Immature Rats through Inactivation of NLRP3 Inflammasome by Inhibiting P2X7R Expression. Int. J. Dev. Neurosci..

[178] Kong F., Ye B., Cao J., Cai X., Lin L., Huang S., Huang W., Huang Z. (2016). Curcumin Represses NLRP3 Inflammasome Activation via TLR4/MyD88/NF-ΚB and P2X7R Signaling in PMA-Induced Macrophages. Front. Pharmacol..

[179] He Q., Jiang L., Man S., Wu L., Hu Y., Chen W. (2018). Curcumin Reduces Neuronal Loss and Inhibits the NLRP3 Inflammasome Activation in an Epileptic Rat Model. Curr. Neurovasc. Res..

[180] Lin T.-Y., Hung C.-Y., Chiu K.-M., Lee M.-Y., Lu C.-W., Wang S.-J. (2022). Neferine, an Alkaloid from Lotus Seed Embryos, Exerts Antiseizure and Neuroprotective Effects in a Kainic Acid-Induced Seizure Model in Rats. Int. J. Mol. Sci..

[181] Samadianzakaria A., Abdolmaleki Z., Faedmaleki F. (2022). The Effect of Valproic Acid and Furosemide on the Regulation of the Inflammasome Complex (NLRP1 and NLRP3 MRNA) in the Brain of Epileptic Animal Model. Brain Res. Bull..

[182] Qin Z., Song J., Lin A., Yang W., Zhang W., Zhong F., Huang L., Lü Y., Yu W. (2022). GPR120 Modulates Epileptic Seizure and Neuroinflammation Mediated by NLRP3 Inflammasome. J. Neuroinflammation.

[183] Xia L., Liu L., Cai Y., Zhang Y., Tong F., Wang Q., Ding J., Wang X. (2021). Inhibition of Gasdermin D-Mediated Pyroptosis Attenuates the Severity of Seizures and Astroglial Damage in Kainic Acid-Induced Epileptic Mice. Front. Pharmacol..

[184] Jimenez-Pacheco A., Diaz-Hernandez M., Arribas-Blázquez M., Sanz-Rodriguez A., Olivos-Oré L.A., Artalejo A.R., Alves M., Letavic M., Miras-Portugal M.T., Conroy R.M. (2016). Transient P2X7 Receptor Antagonism Produces Lasting Reductions in Spontaneous Seizures and Gliosis in Experimental Temporal Lobe Epilepsy. J. Neurosci..

[185] Sabilallah M., Fontanaud P., Linck N., Boussadia B., Peyroutou R., Lasgouzes T., Rassendren F.A., Marchi N., Hirbec H.E. (2016). Evidence for Status Epilepticus and Pro-Inflammatory Changes after Intranasal Kainic Acid Administration in Mice. PLoS ONE.

[186] Pohlentz M.S., Müller P., Cases-Cunillera S., Opitz T., Surges R., Hamed M., Vatter H., Schoch S., Becker A.J., Pitsch J. (2022). Characterisation of NLRP3 Pathway-Related Neuroinflammation in Temporal Lobe Epilepsy. PLoS ONE.

[187] Yue J., Wei Y.J., Yang X.L., Liu S.Y., Yang H., Zhang C.-Q. (2020). NLRP3 Inflammasome and Endoplasmic Reticulum Stress in the Epileptogenic Zone in Temporal Lobe Epilepsy: Molecular Insights into Their Interdependence. Neuropathol. Appl. Neurobiol..

[188] Cristina de Brito Toscano E., Leandro Marciano Vieira É., Boni Rocha Dias B., Vidigal Caliari M., Paula Gonçalves A., Varela Giannetti A., Maurício Siqueira J., Kimie Suemoto C., Elaine Paraizo Leite R., Nitrini R. (2021). NLRP3 and NLRP1 Inflammasomes Are Up-Regulated in Patients with Mesial Temporal Lobe Epilepsy and May Contribute to Overexpression of Caspase-1 and IL-β in Sclerotic Hippocampi. Brain. Res..

[189] Ravizza T., Gagliardi B., Noé F., Boer K., Aronica E., Vezzani A. (2008). Innate and Adaptive Immunity during Epileptogenesis and Spontaneous Seizures: Evidence from Experimental Models and Human Temporal Lobe Epilepsy. Neurobiol. Dis..

[190] Gustin A., Kirchmeyer M., Koncina E., Felten P., Losciuto S., Heurtaux T., Tardivel A., Heuschling P., Dostert C. (2015). NLRP3 Inflammasome Is Expressed and Functional in Mouse Brain Microglia but Not in Astrocytes. PLoS ONE.

[191] Song P., Hu J., Liu X., Deng X. (2019). Increased Expression of the P2X7 Receptor in Temporal Lobe Epilepsy: Animal Models and Clinical Evidence. Mol. Med. Rep..

[192] Löscher W., Klein P. (2021). The Pharmacology and Clinical Efficacy of Antiseizure Medications: From Bromide Salts to Cenobamate and Beyond. CNS Drugs.

[193] Janmohamed M., Brodie M.J., Kwan P. (2020). Pharmacoresistance—Epidemiology, Mechanisms, and Impact on Epilepsy Treatment. Neuropharmacology.

[194] Yu H., He B., Han X., Yan T. (2021). Rufinamide (RUF) Suppresses Inflammation and Maintains the Integrity of the Blood-Brain Barrier during Kainic Acid-Induced Brain Damage. Open Life Sci..

[195] Terrone G., Balosso S., Pauletti A., Ravizza T., Vezzani A. (2020). Inflammation and Reactive Oxygen Species as Disease Modifiers in Epilepsy. Neuropharmacology.

[196] Bialer M., Johannessen S.I., Levy R.H., Perucca E., Tomson T., White H.S. (2013). Progress Report on New Antiepileptic Drugs: A Summary of the Eleventh Eilat Conference (EILAT XI). Epilepsy Res..

[197] Vezzani A., Balosso S., Ravizza T. (2019). Neuroinflammatory Pathways as Treatment Targets and Biomarkers in Epilepsy. Nat. Rev. Neurol..

[198] Albanese V., Missiroli S., Perrone M., Fabbri M., Boncompagni C., Pacifico S., De Ventura T., Ciancetta A., Dondio G., Kricek F. (2023). Novel Aryl Sulfonamide Derivatives as NLRP3 Inflammasome Inhibitors for the Potential Treatment of Cancer. J. Med. Chem..

[199] Zahid A., Li B., Kombe A.J.K., Jin T., Tao J. (2019). Pharmacological Inhibitors of the NLRP3 Inflammasome. Front. Immunol..

[200] El-Sharkawy L.Y., Brough D., Freeman S. (2020). Inhibiting the NLRP3 Inflammasome. Molecules.

[201] Arioz B.I., Tarakcioglu E., Olcum M., Genc S. (2021). The Role of Melatonin on NLRP3 Inflammasome Activation in Diseases. Antioxidants.

[202] Savina T.A., Balashova O.A., Shchipakina T.G. (2006). Effect of Chronic Consumption of Sodium Valproate and Melatonin on Seizure Activity in Krushinskii-Molodkina Rats. Bull. Exp. Biol. Med..

[203] Shen K., Jiang W., Zhang C., Cai L., Wang Q., Yu H., Tang Z., Gu Z., Chen B. (2021). Molecular Mechanism of a Specific NLRP3 Inhibitor to Alleviate Seizure Severity Induced by Pentylenetetrazole. Curr. Mol. Pharmacol..

[204] Shen K., Mao Q., Yin X., Zhang C., Jin Y., Deng A., Gu Z., Chen B. (2018). NLRP3 Inflammasome Activation Leads to Epileptic Neuronal Apoptosis. Curr. Neurovasc. Res..

[205] Missiroli S., Perrone M., Gafà R., Nicoli F., Bonora M., Morciano G., Boncompagni C., Marchi S., Lebiedzinska-Arciszewska M., Vezzani B. (2023). PML at Mitochondria-Associated Membranes Governs a Trimeric Complex with NLRP3 and P2X7R That Modulates the Tumor Immune Microenvironment. Cell Death Differ..

[206] Bellera C.L., Talevi A. (2019). Quantitative Structure-Activity Relationship Models for Compounds with Anticonvulsant Activity. Expert Opin. Drug Discov..

[207] Zhang J., Mu K., Yang P., Feng X., Zhang D., Fan X., Wang Q., Mao S. (2021). Synthesis, Antiepileptic Effects, and Structure-Activity Relationships of α-Asarone Derivatives: In Vitro and in Vivo Neuroprotective Effect of Selected Derivatives. Bioorg. Chem..

[208] Jha M., Alam O., Naim M.J., Sharma V., Bhatia P., Sheikh A.A., Nawaz F., Alam P., Manaithiya A., Kumar V. (2020). Recent Advancement in the Discovery and Development of Anti-Epileptic Biomolecules: An Insight into Structure Activity Relationship and Docking. Eur. J. Pharm. Sci..

[209] Keri R.S., Budagumpi S., Balappa Somappa S. (2022). Synthetic and Natural Coumarins as Potent Anticonvulsant Agents: A Review with Structure-Activity Relationship. J. Clin. Pharm. Ther..

[210] Kwan P., Brodie M.J. (2007). Emerging Drugs for Epilepsy. Expert Opin. Emerg. Drugs.

[211] Bialer M., Johannessen S.I., Kupferberg H.J., Levy R.H., Perucca E., Tomson T. (2007). Progress Report on New Antiepileptic Drugs: A Summary of the Eigth Eilat Conference (EILAT VIII). Epilepsy Res..

[212] Perucca E., French J., Bialer M. (2007). Development of New Antiepileptic Drugs: Challenges, Incentives, and Recent Advances. Lancet Neurol..

[213] Rogawski M.A. (2006). Diverse Mechanisms of Antiepileptic Drugs in the Development Pipeline. Epilepsy Res..

[214] Malawska B., Kulig K. (2008). Brivaracetam: A New Drug in Development for Epilepsy and Neuropathic Pain. Expert Opin. Investig. Drugs.

[215] Kulig K., Więckowski K., Więckowska A., Gajda J., Pochwat B., Höfner G.C., Wanner K.T., Malawska B. (2011). Synthesis and Biological Evaluation of New Derivatives of 2-Substituted 4-Hydroxybutanamides as GABA Uptake Inhibitors. Eur. J. Med. Chem..

[216] Tapia-Abellán A., Angosto-Bazarra D., Martínez-Banaclocha H., de Torre-Minguela C., Cerón-Carrasco J.P., Pérez-Sánchez H., Arostegui J.I., Pelegrin P. (2019). MCC950 Closes the Active Conformation of NLRP3 to an Inactive State. Nat. Chem. Biol..

[217] Coll R.C., Hill J.R., Day C.J., Zamoshnikova A., Boucher D., Massey N.L., Chitty J.L., Fraser J.A., Jennings M.P., Robertson A.A.B. (2019). MCC950 Directly Targets the NLRP3 ATP-Hydrolysis Motif for Inflammasome Inhibition. Nat. Chem. Biol..

[218] Jiang H., He H., Chen Y., Huang W., Cheng J., Ye J., Wang A., Tao J., Wang C., Liu Q. (2017). Identification of a Selective and Direct NLRP3 Inhibitor to Treat Inflammatory Disorders. J. Exp. Med..

[219] Choo B.K.M., Kundap U.P., Faudzi S.M.M., Abas F., Shaikh M.F., Samarut É. (2021). Identification of Curcumin Analogues with Anti-Seizure Potential in Vivo Using Chemical and Genetic Zebrafish Larva Seizure Models. Biomed. Pharmacother..

